# Adaptations to High Salt in a Halophilic Protist: Differential Expression and Gene Acquisitions through Duplications and Gene Transfers

**DOI:** 10.3389/fmicb.2017.00944

**Published:** 2017-05-29

**Authors:** Tommy Harding, Andrew J. Roger, Alastair G. B. Simpson

**Affiliations:** ^1^Department of Biochemistry and Molecular Biology, Centre for Comparative Genomics and Evolutionary Bioinformatics, Dalhousie UniversityHalifax, NS, Canada; ^2^Department of Biology and Centre for Comparative Genomics and Evolutionary Bioinformatics, Dalhousie UniversityHalifax, NS, Canada

**Keywords:** halophile, protozoa, gene duplication, lateral gene transfer, transcriptomics, stress, sodium antiporter, sterol

## Abstract

The capacity of halophiles to thrive in extreme hypersaline habitats derives partly from the tight regulation of ion homeostasis, the salt-dependent adjustment of plasma membrane fluidity, and the increased capability to manage oxidative stress. Halophilic bacteria, and archaea have been intensively studied, and substantial research has been conducted on halophilic fungi, and the green alga *Dunaliella*. By contrast, there have been very few investigations of halophiles that are phagotrophic protists, i.e., protozoa. To gather fundamental knowledge about salt adaptation in these organisms, we studied the transcriptome-level response of *Halocafeteria seosinensis* (Stramenopiles) grown under contrasting salinities. We provided further evolutionary context to our analysis by identifying genes that underwent recent duplications. Genes that were highly responsive to salinity variations were involved in stress response (e.g., chaperones), ion homeostasis (e.g., Na^+^/H^+^ transporter), metabolism and transport of lipids (e.g., sterol biosynthetic genes), carbohydrate metabolism (e.g., glycosidases), and signal transduction pathways (e.g., transcription factors). A significantly high proportion (43%) of duplicated genes were also differentially expressed, accentuating the importance of gene expansion in adaptation by *H. seosinensis* to high salt environments. Furthermore, we found two genes that were lateral acquisitions from bacteria, and were also highly up-regulated and highly expressed at high salt, suggesting that this evolutionary mechanism could also have facilitated adaptation to high salt. We propose that a transition toward high-salt adaptation in the ancestors of *H. seosinensis* required the acquisition of new genes via duplication, and some lateral gene transfers (LGTs), as well as the alteration of transcriptional programs, leading to increased stress resistance, proper establishment of ion gradients, and modification of cell structure properties like membrane fluidity.

## Introduction

Hypersaline environments are habitats for a variety of halophilic microorganisms that are adapted to the often-extreme conditions prevailing in these settings. True halophilic microbes require the presence of salt to grow optimally and several cannot divide at salt concentrations under ~9%, which is around three times the salinity of seawater (Gochnauer et al., 1975; Oren, 2002a; Park et al., 2006, 2007, 2009; Cho et al., 2008; Kunčič et al., 2010; Park and Simpson, 2011; Foissner et al., 2014). Challenges faced by these organisms include ionic stress (especially the toxicity of sodium and chloride ions), osmotic stress, dehydration/desiccation stress (induced by complete evaporation), and reduced solubility of metabolites including nutrients and oxygen. Halophiles have evolved to overcome these constraints by developing adaptations such as amino acid bias in high-salt exposed proteins (Frolow et al., 1996; Paul et al., 2008) and massive synthesis of organic osmolytes (Borowitzka and Brown, 1974; Galinski, 1995; Oren, 2002b).

Compared to halophilic bacteria and archaea, much less information is available regarding the molecular biology and physiology of halophilic microbial eukaryotes. Most of our knowledge comes from the model yeasts *Hortaea werneckii* and *Wallemia ichthyophaga*, and species of the chlorophycean alga genus *Dunaliella*. In *H. werneckii*, higher salinities induce the up-regulation of a persistent transcriptional program for specific genes that is substantially controlled by the mitogen-activated protein kinase (MAPK) Hog1 (Vaupotic and Plemenitaš, 2007). As the downstream effector of a cascade of MAPKs, Hog1 is the central regulator of the high osmolarity glycerol pathway that regulates the expression of various osmoresponsive genes, including those involved in ionic homeostasis, energy metabolism, and protein quality control (Plemenitaš et al., 2008). For example, Hog1 regulates the expression of cation expulsion ATPases, encoded by the *ENA* genes, that maintain low intracellular sodium ion content relative to the extracellular medium (Gorjan and Plemenitaš, 2006). Salts impact membrane fluidity, so adaptation to varying salinities involves adjustment of membrane lipid composition (Russell, 1989); *H. werneckii* maintains a fluid membrane over a wide range of salinities by keeping a low sterol-to-phospholipid ratio and by decreasing both fatty acyl length and the saturation level of phospholipids (Turk et al., 2004, 2007).

When exposed to high salt conditions, microorganisms face another important challenge: oxidative stress. Mitochondria are probably the dominant source of reactive oxygen species (ROS); stress conditions induce an imbalance in the electron transport chain, leading to reverse electron transport, and undesired oxidation of oxygen by complex I (Tomanek, 2015). Several studies in plants indicate that acquisition of salt tolerance might be a consequence of improving resistance to oxidative stress (e.g., Hernández et al., 1995, 2000; Gossett et al., 1996; Gueta-Dahan et al., 1997). Concordantly, the ability of *H. werneckii* to manage oxidative stress appears to be accentuated, since its capability to degrade hydrogen peroxide over a wide range of salinity is as high, or even higher, than that of *Saccharomyces cerevisiae* stressed by exposure to 3% salt (Petrovič, 2006). In addition, the molecular chaperones Hsp70 and Hsp90 are up-regulated at high salt in *H. werneckii* and contribute to control proteins damaged by stress conditions (Vaupotic and Plemenitaš, 2007).

Halophilic yeasts and algae represent only a portion of the diversity of halophilic eukaryotes. A substantial and broad diversity of heterotrophic protists (i.e., protozoa) are known to inhabit extremely hypersaline habitats (see Hauer and Rogerson, 2005; Park et al., 2009; Park and Simpson, 2015), and several of these have been grown in culture at high salinities (exceeding 20% salt; Park et al., 2007, 2009; Cho et al., 2008; Foissner et al., 2014). At present, however, there is virtually no information available on the molecular adaptations of halophilic heterotrophic protists. Although protozoa and fungi are both heterotrophic, most protozoa feed by phagocytosing particles (typically prokaryotes) and thus differ greatly from yeasts, which are osmotrophs. In addition, phagotrophic protozoa are typically not surrounded by a cell wall. These differences between yeasts and protozoa are likely to lead to very different salt adaptation strategies.

The bicosoecid stramenopile *Halocafeteria seosinensis* was first isolated from a 30% salt Korean saltern (Park et al., 2006), and the *Halocafeteria* clade has been frequently observed in hypersaline water samples from various geographic locations (Park and Simpson, 2015). *H. seosinensis* strain EHF34 grows optimally at 15% salt and still divides at 30% salt, but cannot grow at salinities <7.5% (Park et al., 2006). Analysis of its inferred cytoplasmic proteome revealed a molecular signature suggestive of a higher intracellular salt content than in marine protists (Harding et al., 2016). This was also detected in the halophilic heterolobosean *Pharyngomonas kirbyi*, suggesting this property might be typical for halophilic protozoa. At high salt, *H. seosinensis* up-regulates genes whose products are potentially involved in osmolyte synthesis and transport, namely ectoine hydroxylase, amino acid transporters, and myo-inositol transporters, suggesting it might use organic solutes to reach osmotic equilibrium (Harding et al., 2016).

Here we present a broad analysis of *H. seosinensis* transcriptomes generated under moderate and high salt conditions. These conditions are considered with an ecological perspective, i.e., recognizing that the treatments differ not only in salt concentration, but also in other factors that vary according to salinity, notably oxygen availability. We report on the long-term transcriptional program of salt-adapted cells, with an emphasis on genes that were significantly up-regulated at high salt. We also identify gene duplications and probable lateral gene transfer (LGT) events that potentially contributed to the halophilicity of *H. seosinensis*, similarly to previous studies on halophilic yeast and the polyextremophile alga *Galdieria sulphuraria* (Lenassi et al., 2013; Schönknecht et al., 2013; Zajc et al., 2013). Although there are limitations to predicting gene function solely based on sequence information, differential expression studies are extremely helpful in order to flag genes with important physiological roles (e.g., Diray-Arce et al., 2015). With the identification of candidate salt-responsive systems in cell physiology (among thousands of possibilities), credible hypotheses can be proposed that can be experimentally tested in future.

## Materials and methods

### RNA extraction and sequence generation

Transcriptomic sequences from *H. seosinensis* strain EHF34 (Park et al., 2006) were generated and deposited in GenBank as described by Harding et al. (2016). Briefly, RNA was extracted from mid-exponential cultures grown in triplicate in 15 and 30% salt minimal media, and fed with *Haloferax* sp. RNA was extracted using TRIzol (Rio et al., 2010) and treated with Turbo DNAse (Ambion) prior to cDNA library preparation using the TruSeq RNA sample preparation kit version 2 (Illumina) that included a poly-A tail purification step to enrich for eukaryotic messenger RNA (mRNA). *S*amples were sequenced on a HiSeq platform by Génome Québec. Reads were trimmed to remove low-quality sequences using Trimmomatic v. 0.30 (Bolger et al., 2014) and mapped to genomes of food prokaryotes known to be in the culture in order to discard contaminant sequences, using Stampy 1.0.23 (Lunter and Goodson, 2011). Reads were then assembled using Trinity 2.0.2 (Grabherr et al., 2011) and open-reading frames (ORFs) were predicted using TransDecoder (included in the Trinity package). Nucleotide sequences were compared to each other using BLASTN (Altschul et al., 1990) and ORFs sharing identical stretches of at least 50 nucleotides were considered alternative spliced isoforms of the same gene. Genomic data generated by Harding et al. (2016) were used to validate this assignment of isoforms to genes. Finally, to remove sequences belonging to any unknown prokaryotic contaminants present in the cultures (or sequence data), the nucleotide sequences of ORFs were compared to sequences in the NCBI Nucleotide collection (NT) database using BLASTN. Sequences having >100 bp-long regions >90% identical to a prokaryotic sequence were discarded.

### Gene annotation

Predicted proteins were annotated using the eggNOG 4.1 database (Powell et al., 2014) through hidden Markov model searches (*E* < 0.00001) using the hmmsearch program of the HMMER package (Eddy, 1998). Further protein domain characterization was done by interrogating the Pfam 27.0 (Finn et al., 2016) and TIGRFAMs (Haft et al., 2003) databases using hmmsearch, and the NCBI conserved domain database (Marchler-Bauer et al., 2015) using the BLAST algorithm. Proteins were also assigned to KEGG pathways (Kanehisa et al., 2016) by the KEGG Automatic Annotation Server (Moriya et al., 2007) using the representative set for genes through the single-directional best hit method.

In specific cases, putative functions of *H. seosinensis* proteins were investigated further by inspecting multiple sequence alignments for conserved functional residues and by performing phylogenetic analyses. In these instances, searches of the NCBI non-redundant (NR) database and the Marine Microbial Eukaryote Transcriptome Sequencing Project (MMETSP; Keeling et al., 2014) were conducted using BLAST to gather homologous genes. Sequences were aligned using MAFFT 7.205 (Katoh et al., 2002) and resulting alignments were trimmed using BMGE 1.1 (Criscuolo and Gribaldo, 2010). Maximum-likelihood phylogenetic trees were inferred using RAxML 8.1.22 (Stamatakis et al., 2005) with the PROTGAMMALG4X model of amino acid substitution and five starting trees. Bootstrap support was calculated from 100 pseudo replicates.

Protein features such as transmembrane regions and signal peptides were searched for, in order to increase confidence in annotations in cases where these characteristics had been previously reported. Targeting signals were predicted using TargetP 1.1 (Emanuelsson et al., 2000), mitoprot II 1.101 (Claros and Vincens, 1996), and Phobius (Käll et al., 2004). Sequences with predicted signal peptides were investigated further for the presence of the endoplasmic reticulum (ER) retention signals KK, KxK, KDEL, or HDEL at the C-terminus, or RR at the N-terminus. Transmembrane domains were predicted using TMHMM 2.0 (Krogh et al., 2001) and HMMTOP 2.0 (Tusnady and Simon, 2001).

### Differential gene expression assessment

Gene expression at optimal and maximal salt concentrations was quantified using RSEM (Li and Dewey, 2011). Briefly, forward sequence reads from each replicate were mapped to the Trinity assembly using Bowtie 2 v.2.2.4 (Langmead et al., 2009). After removal of ORFs having low read counts in all samples (75th quantile <10 reads), differential expression was assessed using three independent software programs: the empirical Bayesian analysis tool EBSeq following 10 iterations (Leng et al., 2013), DESeq2 (Love et al., 2014) and the limma package (Smyth, 2004; Ritchie et al., 2015) after normalization using the Voom method (Law et al., 2014). *P*-values were corrected for multiple testing using the Benjamini–Hochberg method. ORFs were considered differentially expressed if their posterior probability was above 0.95 (or adjusted *p* < 0.05) and posterior fold change (FC) < 0.5 or >2 (i.e., log_2_FC < −1 or >1).

### Evaluation of prokaryotic contamination based on transcript abundance

To examine the possibility that prokaryotic sequences remained in our dataset after decontamination (Section RNA Extraction and Sequence Generation), sequence reads and assembled contigs were re-processed without any bioinformatic filters for removing prokaryotic sequences, and the abundance of transcripts, including prokaryotic transcripts, was determined as described above. In this analysis, the sequences of prokaryotic origin with highest abundance were highly similar to *Haloferax volcanii* genomic sequences (>95% identity), and thus very likely originated from the supplied food source (*Haloferax* sp.). Importantly, these “confirmed” prokaryotic protein-coding transcripts never had expression levels above 10 transcripts per million (TPM). Since *H. seosinensis* was fed in large excess with *Haloferax* sp. in a mineral medium (i.e., not favoring prokaryotic growth), it is unlikely that transcripts expressed by residual prokaryotes in the cultures would be more abundant. Therefore, the genes presented in this study that were closely related to bacterial sequences were most likely from the *H. seosinensis* genome, since these were more abundantly transcribed by orders of magnitude (>600 TPM; see Section Results). This is further supported by the presence of introns in the corresponding genes (see Section Results). Introns were predicted from genomic sequences as described by Harding et al. (2016).

### Identification of duplicated genes

A local database containing protist protein sequences from the MMETSP dataset and from published genomes (Supplementary Tables 1A,B) was constructed in order to identify recently duplicated genes. *H. seosinensis* sequences were added to this database after selecting the longest isoform for each gene. Following BLASTP comparison using *H. seosinensis* sequences as queries, sequences that were more similar to other *H. seosinensis* sequences than to other eukaryote sequences were classed as “candidate recent duplicates.” For these, additional homologous sequences were gathered by BLASTP comparison against the NR database if the alignment covered >2/3 of the smallest sequence for sequences >30% identical. For each gene cluster, sequences were aligned and trimmed as described in Section Gene Annotation. Preliminary maximum-likelihood phylogenetic trees were inferred using FastTree 1.0.1 (Price et al., 2009). Trees for which *H. seosinensis* sequences clustered in a clade to the exclusion of sequences from other organisms were selected for more in-depth phylogenetic analysis using RAxML, as described in Section Gene Annotation but using 50 independent starting trees for ML tree search. *H. seosinensis* sequences that clustered exclusively in a clade with bootstrap support >50% were considered candidate gene duplicates. As controls, the same analysis was performed on proteins predicted from the genomes of *Dictyostelium discoideum* (GCF_000004695.1), *Guillardia theta* (GCF_000315625.1), *Nannochlorospis gaditana* (GCA_000240725.1), *Salpingoeca rosetta* (GCF_000188695.1), and *Thalassiosira pseudonana* (GCA_000149405.2).

To evaluate whether differentially expressed genes were significantly associated with gene duplication events, we randomly assigned genes to duplicate clusters proportionally to the results (Section Gene Duplication Analysis) obtained given the method described in the previous paragraph. Genes (*n* = 435) were randomly picked from the complete gene dataset (excluding genes with extremely low transcript abundance, *n* = 11,280 genes), and assigned to 153 clusters to obtain the percentage of clusters containing both up- and down-regulated genes. We repeated this random assignment 1,000 times to obtain an average of such clusters.

To determine how common was the duplication of P2X receptor (P2XR) genes in protists, the genomes of 15 organisms (Supplementary Table 2) and 359 transcriptomes sequenced during the MMETSP (excluding dinoflagellates, whose genomes are known to contain many highly duplicated genes) were searched for sequences homologous to *H. seosinensis* P2XR-related sequences (*E* < 0.00001). Homologs harvested using *H. seosinensis* P2XR sequences as queries were used to interrogate the dataset from their respective species of origin in order to recover more gene duplicates (excluding sequences >90% identical).

### Gene enrichment analysis

The relative abundance of genes assigned to Clusters of Orthologous Group of proteins (COG) was analyzed using STAMP v.2.1.3 (Parks et al., 2014) to determine if they were enriched in differentially expressed genes or duplicated genes. Significant variations in proportions were assessed with the hypergeometric test, followed by multiple-test correction by the Benjamini–Hochberg method. The following COG classes (which contained very few genes, or genes without obvious biological significance or known function) were removed prior to the analysis to decrease their influence on the multiple-test correction: “Cell motility,” “Defense mechanisms,” “Unknown function,” “General predictions only,” “Nuclear structures,” and “No hits found.”

## Results

### Gene expression analysis

**Figure 1:**
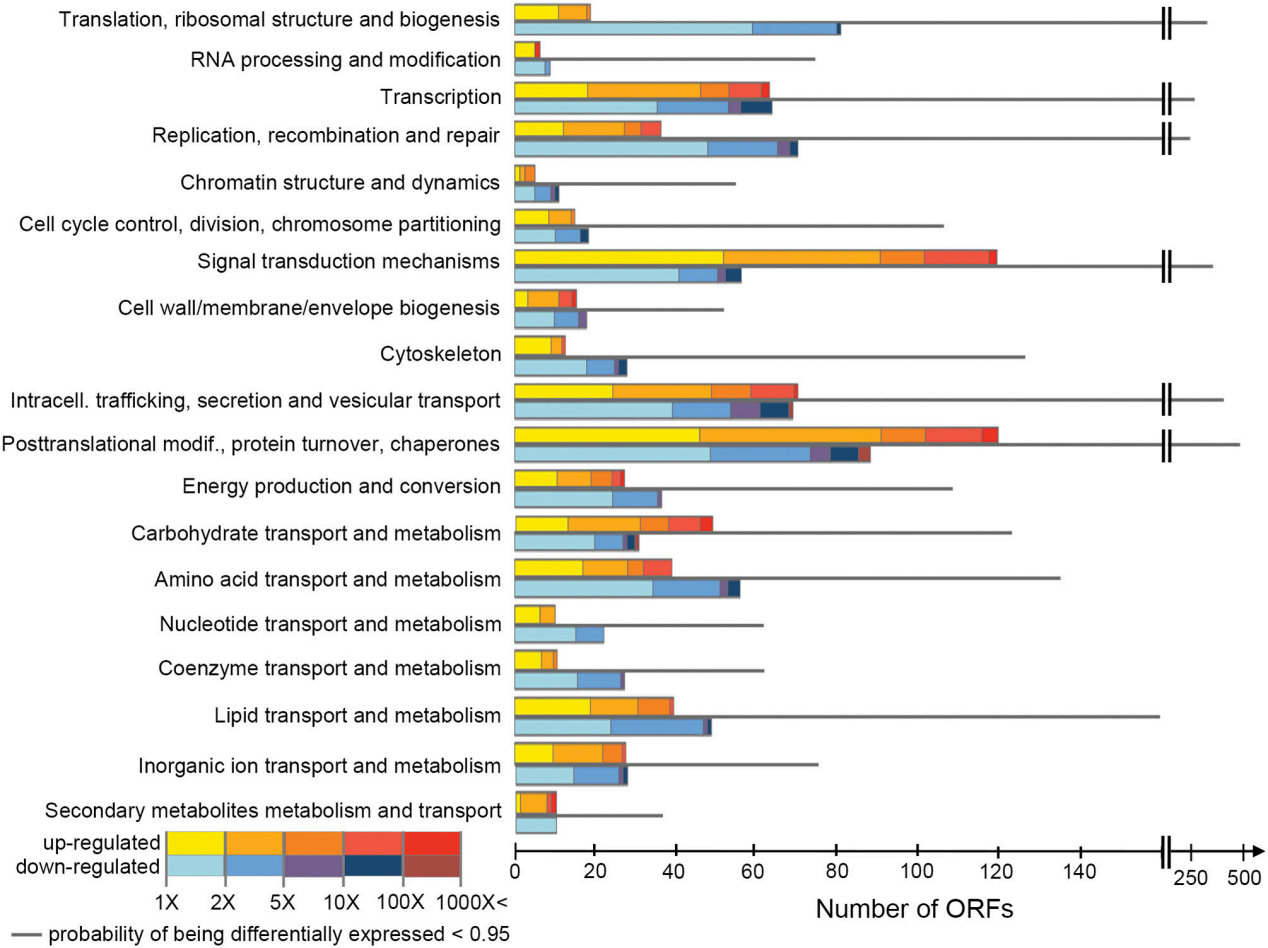
**Numbers of ORFs assigned to each COG class, showing their expression values**. For each class, the first and the last bars indicate the number of ORFs up-regulated at high salt and down-regulated at high salt, respectively (posterior probability of being differentially expressed, PPDE > 0.95). Their posterior fold change is indicated by the color scale. The thinner gray bar in the middle shows the number of ORFs in the class with PPDE < 0.95.

*H. seosinensis* expressed 16,852 non-redundant ORFs corresponding to 12,020 genes. Of these, 1,656 ORFs were discarded during differential expression assessment due to low expression in all conditions (see Section Differential Gene Expression Assessment). EBSeq flagged 2,871 ORFs as being differentially expressed, of which 62% were up-regulated at high salt and 38% were down-regulated (Figure 1). BLASTP searches indicated that 45% of these ORFs had no homolog in the NR database (*E*-value cutoff = 0.00001). DESeq2 and limma detected 3,265 and 2,882 differentially expressed ORFs, respectively. There was good agreement between the analyses: 2,418 ORFs were identified as differentially expressed by all three analyses, and the great majority of the ORFs that were flagged as differentially expressed by EBSeq were also identified by limma and DESeq2 (87 and 90%, respectively). For the sake of brevity, only the results from EBSeq (which were the most conservative) are given in the text; predictions from limma and DESeq2 can be found in tables and figures.

**Figure 2:**
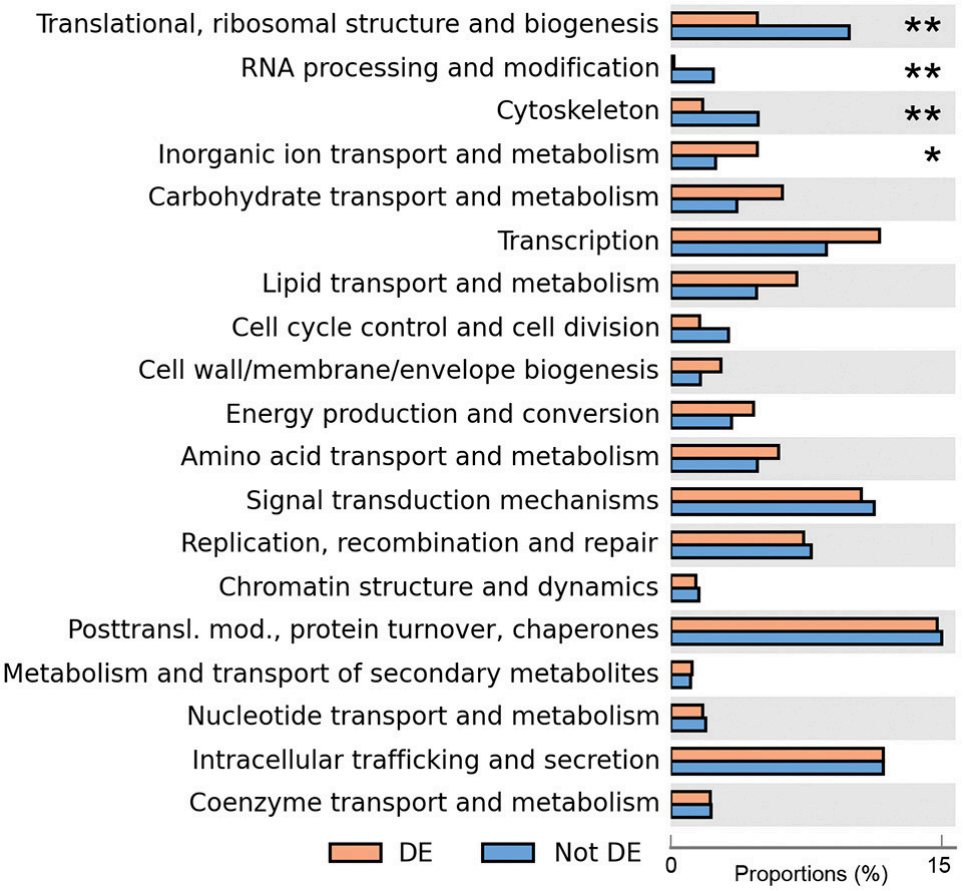
**Enrichment analysis of differentially expressed genes**. The proportions of all the differentially expressed genes that are assigned to each class are shown in orange, and proportions of all non-differentially expressed genes are in blue. The number of asterisks indicates adjusted *p*-values after multiple-test correction using the Benjamini–Hochberg method (^**^adjusted *p* < 0.01, ^*^adjusted *p* < 0.05).

COG classes containing genes involved in metabolism and transport of inorganic ions were significantly enriched in differentially expressed genes (although with relatively low support, adjusted *p* = 0.050), while classes containing genes involved in translation, RNA processing and cytoskeleton were significantly under-represented amongst differentially expressed genes (adjusted *p* < 7.3 × 10^−3^, Figure 2). This suggested that, while the cohort of core genes involved in basic cellular functions tended to not be differentially expressed, genes involved in ion homeostasis as a group responded to a variation in extracellular salinity.

### Gene duplication analysis

The gene duplication analysis revealed 494 clusters containing 1,652 genes. The bulk of these, 317 clusters encompassing 1,086 genes, contained only *H. seosinensis* sequences (i.e., no homologs were detected in other organisms), while 153 clusters contained 435 genes where *H. seosinensis* sequences clustered together (with bootstrap support >50%) to the exclusion of all other homologous sequences gathered from the local protist database and the NR database.

**Figure 3:**
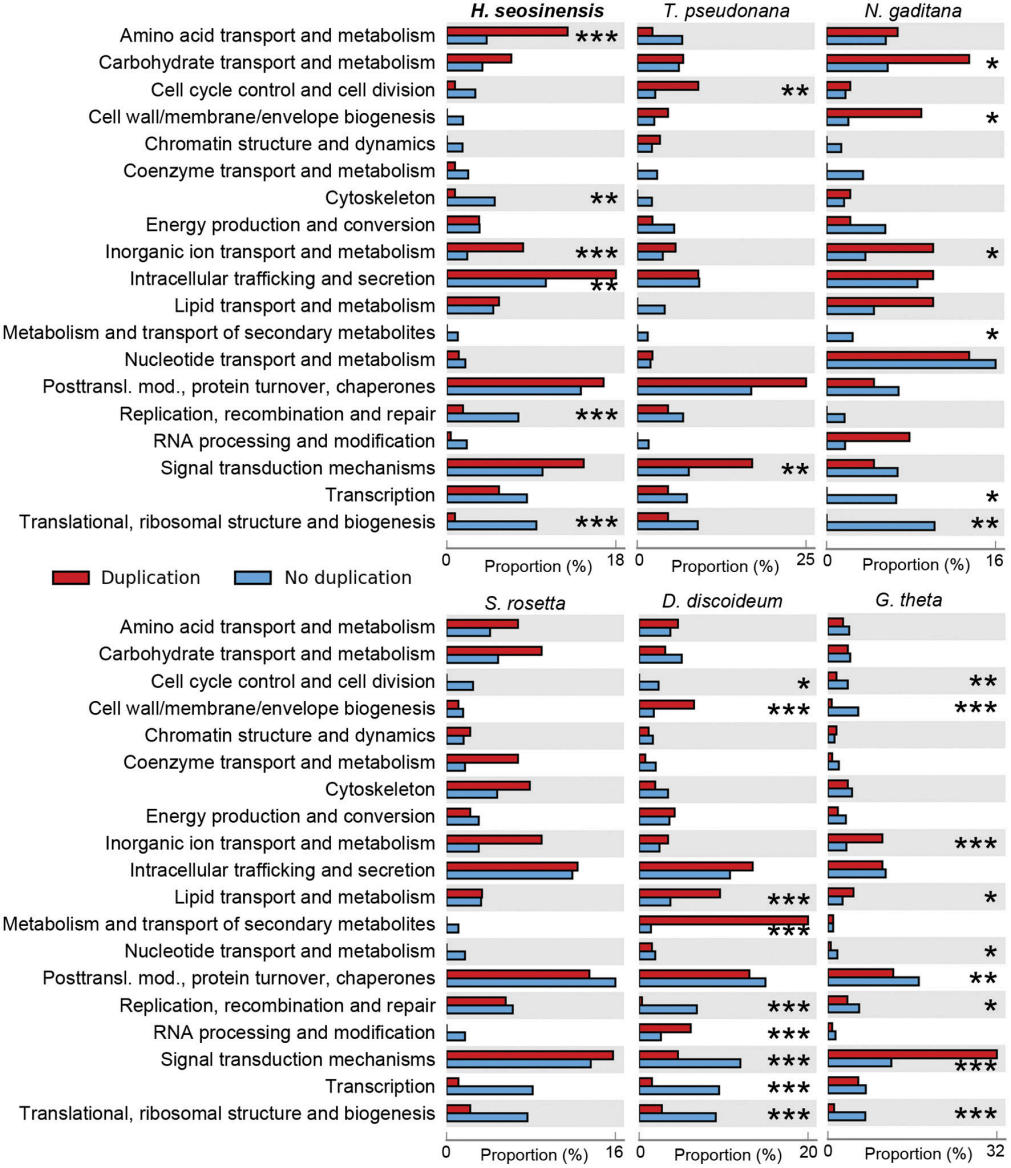
**Gene duplication enrichment analysis performed on the transcriptome of *H. seosinensis*, and the genomes of representative non-halophilic protists (*Thalassiosira pseudonana*, *Nannochloropsis gaditana, Salpingoeca rosetta*, *Dictyostelium discoideum*, and *Guillardia theta*)**. The proportions of all duplicated genes that are assigned to each COG class are shown in red. The proportions of all the remaining genes are in blue. The number of asterisks indicates the false discovery rate after multiple test correction using the Benjamini–Hochberg method (^***^adjusted *p* < 0.001, ^**^adjusted *p* < 0.01, ^*^adjusted *p* < 0.05).

After removing uninformative COG categories (see Section Gene Enrichment Analysis), 230 genes were detected as duplicated in *H. seosinensis*, from a total of 4,283 genes assigned to these categories (6%). Enrichment analysis showed that COG categories representing housekeeping genes (“Translational, ribosomal structure, and biogenesis,” “Replication, recombination and repair,” and “Cytoskeleton”) were depleted of duplicated genes in *H. seosinensis* (adjusted *p* < 4.5 × 10^−3^, Figure 3). Similar results were observed in the genomes of *N. gaditana, G. theta*, and *D. discoideum*. Conversely, categories including genes involved in metabolism and transport of amino acids and inorganic ions were enriched in duplicated genes, as were genes involved in intracellular trafficking of metabolites like phospholipids (adjusted *p* < 4.3 × 10^−3^); enrichment of the first and the latter of these were unique to *H. seosinensis* among the six genomes considered; the second was shared with *N. gaditana* and *G. theta*. The enrichment analysis of duplicated genes indicated that core genes seemed more evolutionarily “stable” compared to metabolic genes, echoing the enrichment analysis of differentially expressed genes, where core genes involved in basic cellular functions were transcriptionally steadier (see above). The same kind of principle was described in fungi, where genes essential in growth processes had more stable copy numbers and expression, while accessory genes were more “volatile” in this regard (Wapinski et al., 2007).

Salt-responsive genes were over-represented among the products of duplication events, highlighting the important potential of this mechanism to contribute to adaptation to high salt environments. A significantly high proportion (43%) of gene duplicates were differentially expressed (hypergeometric test, *p* < < 0.001). Furthermore, gene duplicate clusters were enriched in clusters containing both up- and down-regulated genes (18% of clusters, compared to a random expectation of 3.3 ± 1.3% (average ± 1 standard deviation), see Section Identification of Duplicated Genes).

### Ion homeostasis

Maintaining a steep ion gradient (especially of Na^+^) is key to survival for halophiles, especially for bacterivores like *H. seosinensis* that feed on “salt-in” microbes like *Haloferax* sp. for which the intracellular salt content varies with the extracellular salinity. *H. seosinensis* expressed two genes (ORFs m.11942 and m.85102) that were related to the plasma membrane Na^+^/H^+^ antiporter Salt-Overly-Sensitive 1 (SOS1). Importantly, one of them, m.85102, was 2.7-fold upregulated at high salt (increase from 8.7 TPM at 15% salt to 29.5 TPM at 30% salt, PPDE = 0.99, DESeq2 adjusted *p* = 1.9 × 10^−7^, voom-limma adjusted *p* = 0.0005). As in SOS1, these two proteins in *H. seosinensis* had a transmembrane N-terminal region homologous to the NhaP domain (PFAM00999) that contained aspartate and arginine residues essential for ion binding and translocation (Supplementary Figure 1; Hellmer et al., 2003), and a C-terminal region related to cyclic nucleotide binding domain (CNBD, PFAM00027). These sequences also encoded predicted signal peptides, suggesting that they act somewhere along the secretory pathway, i.e., at the plasma membrane and/or in vacuoles (in which case they might pump sodium into vacuoles for later removal from the cell). Na^+^/H^+^ antiporters are key contributors to salt resistance in plants, where SOS1 contributes to Na^+^ expulsion from the cytosol, including in the salt-resistant halophyte *Thellungiella salsuginea* (Blumwald et al., 2000; Oh et al., 2009). The halophilic alga *D. salina* also increases the expression of a Na^+^/H^+^ antiporter as a response to increased salinity (Katz et al., 1992). Interestingly, the *ENA* genes responsible for sodium homeostasis in halophilic yeasts were not detected in *H. seosinensis*.

As noted above, the class “Ion transport and metabolism” was enriched in gene duplicates (adjusted *p* = 5.8 × 10^−5^, Figure 3). Proteins encoded by these genes were related to various ion transporters and channels, for example, type IIC Na^+^/K^+^-ATPases (TIGR01106, Supplementary Figure 2), type IIB calcium-translocating ATPases (TIGR01517, Supplementary Figure 3), chloride channels (CD03685, Supplementary Figure 4) and magnesium transporters (TIGR00400, Supplementary Figure 5).

### Oxygen availability affected gene expression

At 30% salt, oxygen is theoretically 2.6× less soluble compared to 15% salt (Battino et al., 1983; Sherwood et al., 1991). Concordantly, oxygen limitation at high salt appears to have led to partial repression of respiration-related genes in *H. seosinensis*, which was compensated for by the upregulation of some fermentation genes. This indicates that adapting to lower oxygen availability is part of the response to high salt in this species.

At high salt, transcripts encoding subunit E1beta of pyruvate dehydrogenase and some enzymes of the Krebs cycle (isocitrate dehydrogenase and malate dehydrogenase) were differentially expressed; the most affected being repressed up to 2.5-fold (Table 1). Furthermore, enzymes involved in generating the electron carriers, especially those involved in porphyrin and ubiquinone biosynthesis, tended to be more than 2-fold repressed at high salt (Table 1).

**Table 1:** **Differentially expressed genes involved in respiration and fermentation in *Halocafeteria seosinensis***.

**ORF names**	**Abundance (TPM)**		**EBSeq**		**DESeq2**		**VOOM-LIMMA**		**Annotation**
	**15% salt**	**30% salt**	**PPDE**	**Post fold change**	**Adjusted *p*-value**	**log_2_FC**	**Adjusted *p*-value**	**log_2_FC**	
m.87365	291.8	171.8	1.00	0.45	9.9E-08	−1.14	0.006	−1.13	pyruvate dehydrogenase, E1 beta
**KREBS CYCLE**									
m.75687	826.4	434.5	1.00	0.40	9.3E-10	−1.30	0.004	−1.30	malate dehydrogenase
m.29230	166.1	86.6	0.98	0.39	2.0E-06	−1.32	0.008	−1.31	isocitrate dehydrogenase, regulatory subunit
**PORPHYRIN BIOSYNTHESIS**									
m.63860	86.1	49.7	1.00	0.44	1.9E-14	−1.17	0.002	−1.15	uroporphyrinogen decarboxylase
m.60804	125.5	57.9	0.99	0.35	1.1E-06	−1.48	0.007	−1.49	ferrochelatase
m.74554	102.9	28.9	1.00	0.21	4.0E-29	−2.20	0.000	−2.19	*Cox15*
m.34054	73.9	43.9	1.00	0.46	1.4E-08	−1.09	0.004	−1.08	cytochrome *c* heme lyase
**UBIQUINONE SYNTHESIS**									
m.3998	34.7	15.0	1.00	0.33	3.4E-16	−1.59	0.001	−1.59	*Coq6*
m.57053	26.9	16.2	1.00	0.47	0.0001	−1.07	0.014	−1.05	*Coq5*
**FERMENTATION**									
m.80901	12.3	36.5	1.00	2.26	1.5E-20	1.18	0.001	1.21	NADH-dependent fumarate reductase
m.58901	4.1	11.4	1.00	2.08	0.0065	1.03	0.025	1.20	mitochondrial lactate dehydrogenase

*TPM, averaged transcripts per million; PPDE, Probability of being Differentially Expressed; Post Fold Change, posterior fold change (30% over 15% salt); log_2_FC, log_2_ fold change (30% over 15% salt)*.

Concordantly, certain genes potentially involved in fermentation were up-regulated at high salt. Although glycolytic enzymes were not differentially expressed, soluble NADH-dependent fumarate reductase (2.3-fold upregulation) and mitochondrial lactate dehydrogenase (2.1-fold upregulation) had noticeably higher expression at high salt.

### Signal transduction

Grown in two different salt concentrations, *H. seosinensis* differentially expresses genes typically acting in the G-protein pathway and in cyclic nucleotide signaling, various kinases, P2X receptors, and transcription factors involved in stress responses, such as sirtuins and heat shock factors.

**Figure 4:**
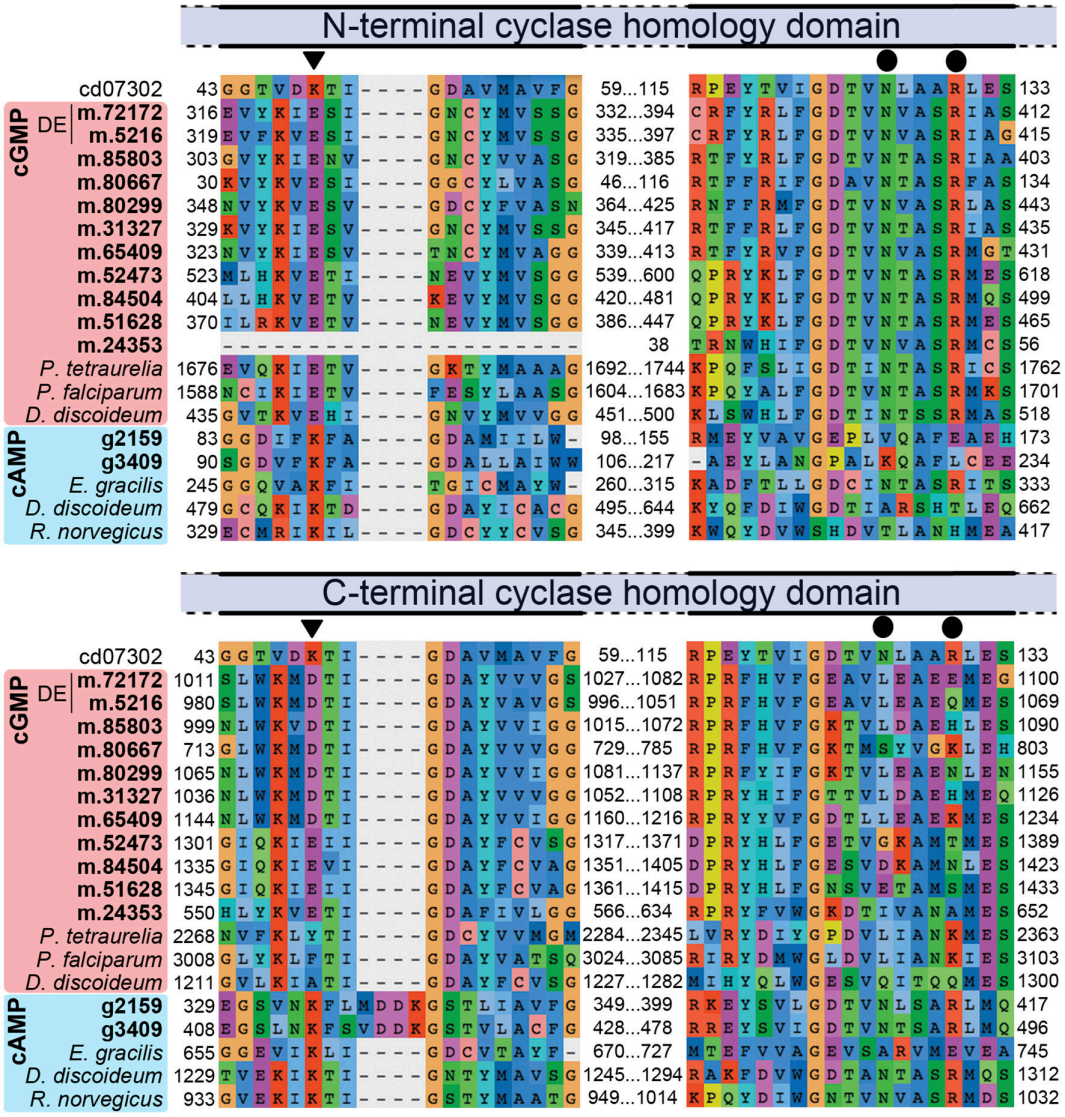
**Partial alignment of nucleotide cyclase sequences showing conservation of residues specific for adenine (K) or guanine (E, triangles on top of alignment) and residues required for catalytic activity (N and R, circles)**. The alignment was generated using the Conserved Domain Database sequence for the cyclase homology domain (CD07302) and both N-terminal and C-terminal domains of *H. seosinensis* sequences (in bold; “DE” indicates sequences that were up-regulated at high salt; m.24353 is a 5′ partial ORF) as well as guanylate cyclase from *Paramecium tetraurelia* (CAB44361.1), *Plasmodium falciparum* (CAD52725.2) and *Dictyostelium discoideum* (CAB42641.1), and adenylate cyclase from *Euglena gracilis* (BAB85619.1), *Dictyostelium discoideum* (Q03100.2), and *Rattus norvegicus* (AAA40682.1). The C-terminal domains in *H. seosinensis* cGMP-specific sequences did not contain these crucial residues, suggesting they were not catalytic domains.

High salt adaptation appears to involve cyclic nucleotide signaling, especially *via* cyclic guanosine monophosphate (cGMP). Some 10 genes encoding membrane-localized proteins related to guanylate cyclase (GC), each containing two class III cyclase catalytic domains (PFAM00211), were expressed in *H. seosinensis*. Two of these genes were markedly up-regulated at high salt (8.9- and 100-fold increase, Table 2). The specificity for guanine in *H. seosinensis* cyclase enzymes was identified based on conserved residues in the purine-binding pocket (Figure 4; Baker and Kelly, 2004). Involvement of cyclic nucleotides as important signaling molecules during salt adaptation was also supported by the differential expression of cyclic nucleotide phosphodiesterases (PDE), the antagonists of nucleotide cyclases. *H. seosinensis* expressed more than 10 proteins encoding a cyclic nucleotide phosphodiesterase domain (PFAM00233), including two that were up-regulated at high salt (4.4- and 4.6-fold, Table 2).

**Table 2:** **Differentially expressed genes involved in cyclic nucleotide signaling in *Halocafeteria seosinensis***.

**ORF names**	**Abundance (TPM)**		**EBSeq**		**DESeq2**		**VOOM-LIMMA**	
	**15% salt**	**30% salt**	**PPDE**	**Post fold change**	**Adjusted *p*-value**	**log_2_FC**	**Adjusted *p*-value**	**log_2_FC**
**GUANYLATE CYCLASES**								
m.5216	0.31	40.70	1.00	100.49	NA	6.53	0.0002	6.72
m.72172	0.30	3.39	1.00	8.85	4.2E-13	3.00	0.0019	3.10
**CYCLIC NUCLEOTIDE PHOSPHODIESTERASES**								
m.89581	0.48	2.87	1.00	4.63	8.3E-05	2.06	0.0134	2.11
m.43482	5.38	30.63	1.00	4.38	2.6E-33	2.12	0.0004	2.14

*TPM, averaged transcripts per million (at 15 or 30% salt); PPDE, Probability of being Differentially Expressed; Post Fold Change, posterior fold change (30% over 15% salt); log_2_FC, log_2_ fold change (30% over 15% salt); NA, not available due to an extreme count outlier in one of the samples*.

The importance of signaling cascades was also indicated by the up-regulation at high salt of many kinases and phosphatases (Supplementary Table 3), including 2 genes related to sensory hybrid kinases encoding Cyclase/Histidine kinase-Associated Sensory Extracellular domains (PFAM03924; m.13308, 32-fold increase and m.13214, 4.5-fold increase), and kinases dependent on mitogen (seven genes with 2.1- to 19-fold over-expression), calcium (five genes with 2.2- to 20-fold over-expression) or calmodulin (one gene with 3.0-fold over-expression), and other serine/threonine protein kinases (five genes with 2.1- to 186-fold over-expression), many of which arose from gene duplication (seven and four clusters for kinases and phosphatases, respectively).

Many genes related to various transcription factors were up-regulated at high salt (Supplementary Table 4) including basic leucine zipper (BZIP) domain-containing factors (PFAM00170 and PFAM07716; 2.1- to 40-fold increase), silent information regulator proteins (sirtuins, CD01410; 3.8- to 15-fold increase), transcription factors of the Myb superfamily (PFAM00249 and PFAM13921; 2.5- to 4.7-fold increase), and factors encoding the AP2 DNA-binding domains (PFAM00847; 14- and 79-fold increase). Interestingly, *H. seosinensis* also over-expressed three genes containing heat-shock factor-type DNA-binding domains (PFAM00447; 2.1- to 3.6-fold increase) that can potentially be linked to the up-regulated chaperones discussed below.

**Figure 5:**
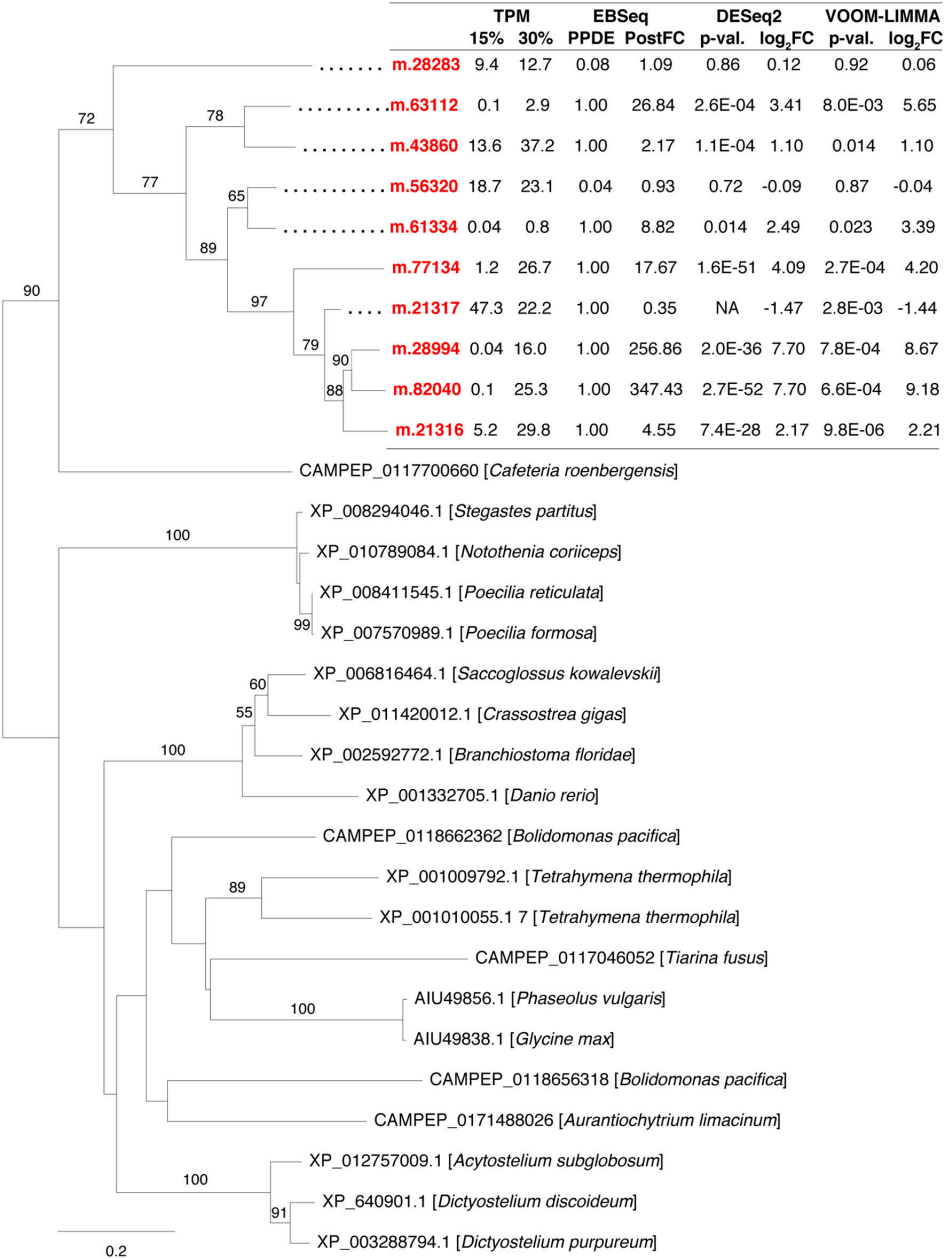
**Maximum-likelihood phylogenetic tree for gene duplication cluster encoding G-protein coupled receptors**. Bootstrap values (>50%) are indicated on branches. The scale bar indicates the expected substitutions/site. The *H. seosinensis* sequences are in red, with expression values indicated: TPM, averaged transcript per million at 15 or 30% salt; PPDE, Posterior Probability of being Differentially Expressed; PostFC, Posterior Fold Change calculated by EBSeq; *p*-val., adjusted *p*-value; log_2_FC, log_2_ fold change calculated either by DESeq2 or voom-limma; NA, not available due to an extreme count outlier in one of the samples.

Duplication of salt-responsive genes indicated that this mechanism probably contributed to *H. seosinensis* high-salt adaptation. For example, *H. seosinensis* up-regulated several genes related to G protein-coupled receptors (GPCRs), sensors of extracellular conditions that were homologous to domains of the *Dictyostelium* slime mold cAMP receptor (PFAM05462) and the membrane region of the Frizzled/Smoothened family (PFAM01534); these genes all possibly originated from gene duplication events (Figure 5). These duplicated genes had contrasting expression levels (from 2.8-fold decrease to 350-fold increase). Three were effectively activated at high salt, as their average transcript level rose from <1.2 TPM at 15% salt to >15 TPM at high salt. Typically, GPCR sequence conservation tends to be low, with an average of <25% pairwise identity between members of the same family (Oliveira et al., 1999). As a result, we could not definitely assign a particular stimulus to each of these GPCRs. However, based on their expression profile and their dynamic recent evolution, many of these genes are likely important for long-term salt adaptation.

**Figure 6:**
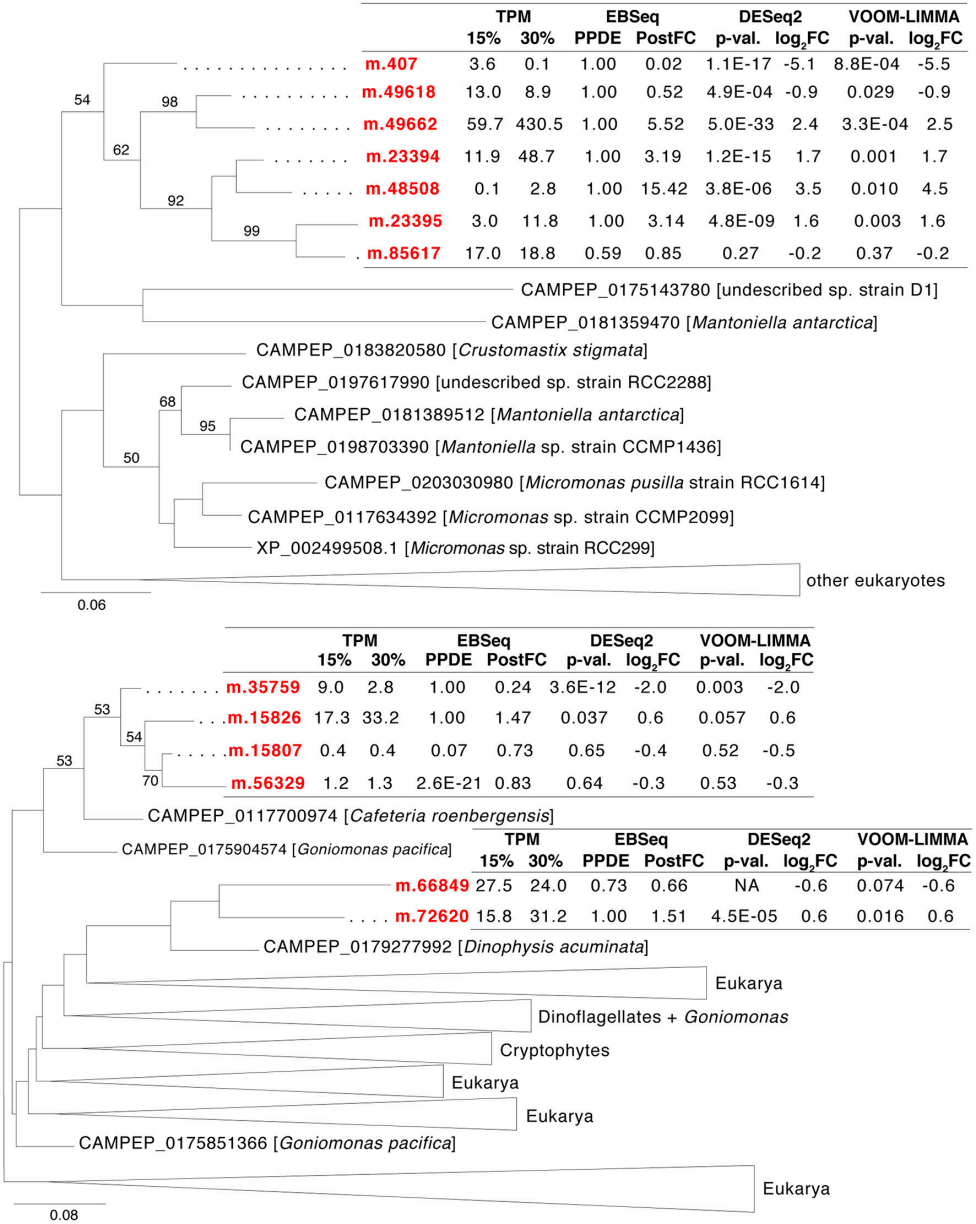
**Maximum-likelihood phylogenetic trees for gene duplication clusters encoding P2X receptors**. Bootstrap values (>50%) are indicated on branches. The scale bar indicates the expected substitutions/site. The *H. seosinensis* sequences are in red, with expression values indicated: TPM, averaged transcript per million at 15 or 30% salt; PPDE, Posterior Probability of being Differentially Expressed; PostFC, Posterior Fold Change calculated by EBSeq; *p*-val., adjusted *p*-value; log_2_FC, log_2_ Fold Change calculated either by DESeq2 or voom-limma; NA, not available due to an extreme count outlier in one of the samples.

Furthermore, *H. seosinensis* expressed 13 genes related to P2X receptors (P2XR, PFAM00864), of which 11 possibly stemmed from recent gene duplication events (Figure 6), including four that were up-regulated (3.1- to 15-fold over-expression, including one with high transcript abundance: m.49662 with 429 TPM at 30% salt) and two that were down-regulated (4.2- and 42-fold repression). P2XR are known as ATP-gated cation channels involved in signaling. However, although the overall structure of P2XR were conserved in *H. seosinensis* sequences (i.e., cytoplasmic N-terminal tail, a longer C-terminal tail, and an extracellular domain delimited by two transmembrane domains), residues binding ATP were not conserved or partially conserved, suggesting these channels were insensitive to ATP (Supplementary Figure 6). However, the protein kinase C (PKC) consensus sequence (Tx[K/R]; Wen and Evans, 2009) was conserved in all of *H. seosinensis* sequences, except in two for which the phosphorylated threonine was substituted by serine (that can potentially be phosphorylated) or alanine, suggesting that some of these channels could be regulated by cytoplasmic PKC as observed for the vertebrate P2XR.

The 13 P2X genes in *H. seosinensis* group into three independent clades, reflecting separate clusters of duplication events (Figure 6, the percentage identity threshold was relaxed to 20% to gather more homologs). In EggNOG, P2XR are assigned to the class “Intracellular trafficking, secretion and vesicular transport” that was enriched in duplicated genes in *H. seosinensis* (adjusted *p* = 4.3 × 10^−3^, Figure 3). By representing 31% of duplicated genes in this class, P2XR contributed substantially to this enrichment. Investigation of the genomes of 15 organisms plus 359 transcriptomes sequenced during the MMETSP indicated that, although duplication of P2XR-related genes occurred in several organisms, high numbers of duplicates per genome are rather uncommon. P2XR-related sequences were detected in seven (47%) of the genomes and 150 (42%) MMETSP transcriptomes, but only eight (2%) protists expressed more than ten P2XR-related proteins (*Pyramimonas parkeae, Pseudo-nitzschia fraudulenta, Chrysochromulina ericina, Chrysochromulina polylepis, Chrysochromulina rotalis, Mesodinium pulex, Dolichomastix tenuilepis*).

### Stress response

High salt induced the up-regulation of an arsenal of genes that are involved in protecting a wide diversity of molecules against oxidative stress, especially proteins and lipids. Some of these were amongst the most highly transcribed genes in the high salt condition, and they are inferred to function in several different cellular compartments. Proteins encoded by these genes either contributed to directly neutralize ROS (e.g., superoxide dismutase and peroxidase), were involved in repairing and protecting cellular components affected by ROS (e.g., glutathione-dependent enzymes and chaperones such as heat shock proteins), or were involved in controlling potential sources of ROS (e.g., quinone oxidoreductase).

Two highly expressed and up-regulated genes encoded superoxide dismutase (SOD, 2.7-fold increase, 1,861 TPM at 30% salt, corresponding to rank 97), which catalyze the dismutation of superoxide radicals, and peroxidase (18.4-fold increase, 3,986 TPM at 30% salt, rank 11), which reduces H_2_O_2_ to water (Table 3). The sequence related to SOD (m.9318) was predicted to encode a cytosolic enzyme, based on phylogenetic analysis and signal prediction (Supplementary Figure 7). Analysis of conserved residues indicated that the enzyme depends on manganese as a co-factor (Supplementary Figure 8), which is rarely documented for cytosolic SODs (Wilkinson et al., 2006; Krueger et al., 2015).

**Table 3:** **Differentially expressed genes involved in neutralizing ROS in *Halocafeteria seosinensis***.

**ORF names**	**Abundance (TPM)**		**EBSeq**		**DESeq2**		**VOOM-LIMMA**	
	**15% salt**	**30% salt**	**PPDE**	**Post fold change**	**Adjusted *p*-values**	**log_2_FC**	**Adjusted *p*-values**	**log_2_FC**
**SUPEROXIDE DISMUTASE**								
m.9318	551.34	1871.39	1.00	2.65	5.8E-06	1.38	0.01	1.42
**PEROXIDASE**								
m.79082	161.47	4003.99	1.00	18.42	3.4E-27	4.04	0.001	4.29
**CYTOSOLIC GLUTATHIONE TRANSFERASES**								
m.57692	108.16	1285.73	1.00	9.06	1.8E-18	3.08	0.001	3.26
m.69131	115.37	583.75	1.00	3.90	2.9E-18	1.95	0.001	1.97
**MICROSOMAL GLUTATHIONE TRANSFERASE**								
m.21576	306.61	1069.01	1.00	2.66	0.0001	1.37	0.02	1.43
**GLUTAREDOXINS**								
m.39259	8.05	46.69	1.00	4.75	6.6E-10	2.17	0.002	2.27
m.72582	222.23	825.45	1.00	2.81	0.0006	1.44	0.02	1.56
**PEROXIREDOXIN 6**								
m.14632	60.92	615.54	1.00	7.74	6.9E-24	2.89	0.0005	2.99

*TPM, averaged transcripts per million; PPDE, Probability of being Differentially Expressed; Post Fold Change, posterior fold change (30% over 15% salt); log_2_FC, log_2_ fold change (30% over 15% salt)*.

**Figure 7:**
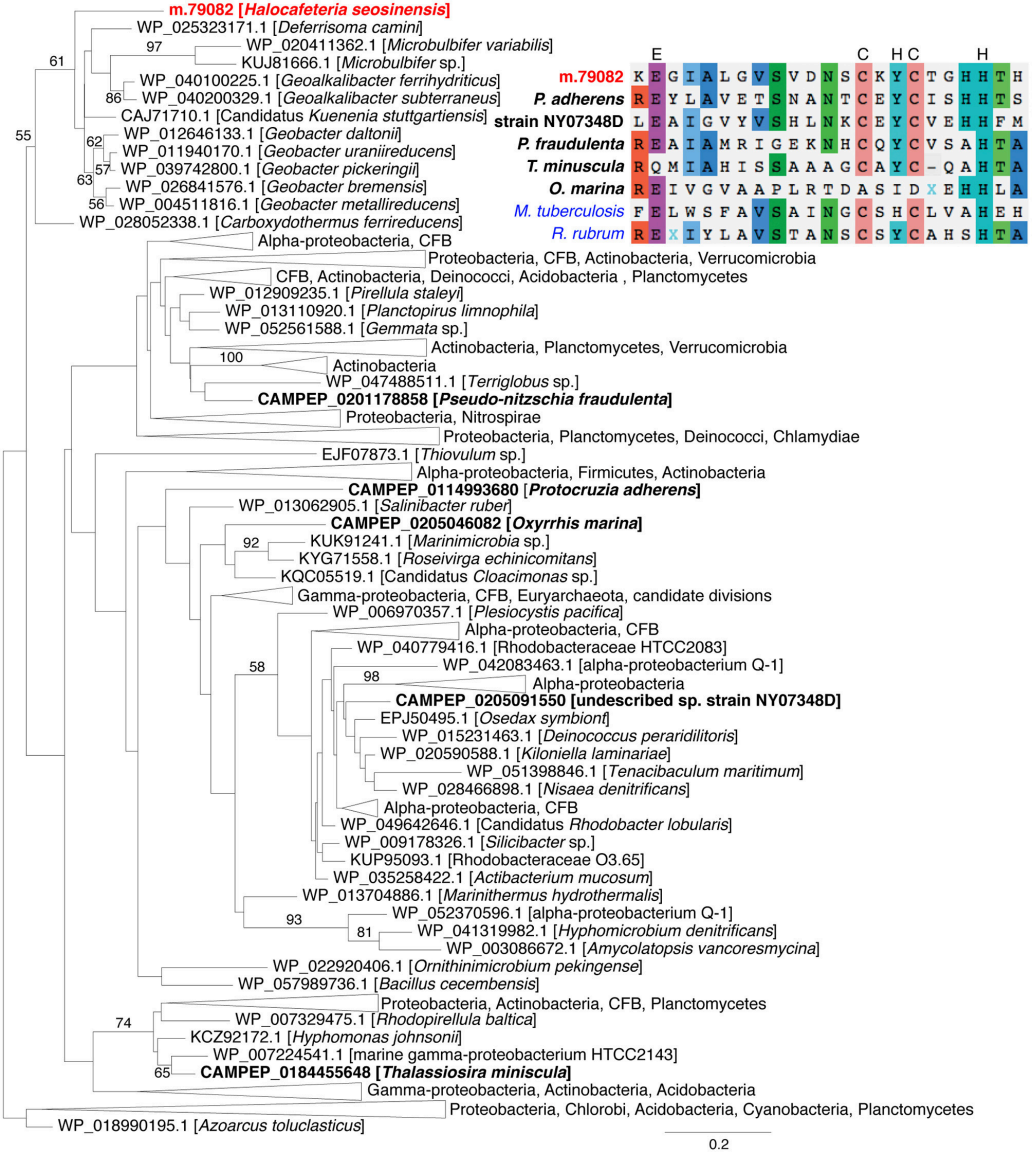
**Maximum-likelihood phylogenetic tree for peroxidase showing the relatedness of eukaryotic sequences (in bold, *H. seosinensis* in red) to prokaryotic sequences**. Bootstrap values (>50%) are indicated on branches. The scale bar indicates the expected substitution rate/site. At the top right, partial alignment of peroxidase sequences from the eukaryotes included in the tree and *Mycobacterium tuberculosis* AhpD protein (in blue, ALB19631), for which essential residues for catalytic activity were determined (displayed on top of the alignment) and the *Rhodospirillum rubrum* AhpD sequence (in blue, 2OUW), for which a crystal structure is available. The partial alignment corresponds to residues 117–139 of *M. tuberculosis* AhpD.

The enzyme related to peroxidase (ORF m.79082) was affiliated with a family of uncharacterized peroxidase-related bacterial enzymes (TIGR01926) and possessed all the residues, except one, shown to catalyze the oxidation of peroxide in the closest characterized enzyme, *Mycobacterium tuberculosis* alkylhydroperoxidase AhpD (Figure 7; Koshkin et al., 2003). Interestingly, a phylogeny showed m.79082 clustered with proteobacterial sequences to the exclusion of all homologous sequences from eukaryotes harvested from the MMETSP dataset (Figure 7), consistent with LGT from a bacterium. The presence of an intron in the 5′-untranslated region (UTR; Supplementary Figure 9A) and the extremely high expression (3,986 TPM) of this gene strongly support m.79082 being in the *H. seosinensis* genome, and not a contaminating bacterial sequence (see Section Evaluation of Prokaryotic Contamination Based on Transcript Abundance). This gene was not detected in *C. roenbergensis*, the closest sister species of *H. seosinensis* for which molecular data are available, it is thus possible that it was acquired relatively recently in the *H. seosinensis* lineage, maybe co-incidentally with adaptation to a halophilic lifestyle. Alternatively, this gene could have been lost in *C. roenbergensis* or not expressed under the growth conditions experienced during the MMETSP data generation.

Enzymes involved in the glutathione-dependent detoxification system were also strongly up-regulated at high salt (Table 3). These included dithiol glutaredoxins, which are responsible for the reduction of protein disulfides and glutathione-protein mixed disulfides (2.8- and 4.8-fold increase), and glutathione transferases (GT) acting on lipophilic substrates. The latter included ORF m.21576 (2.7-fold increase, 1,064 TPM at 30% salt, rank 420), which was related to microsomal GT and that originated from a gene duplication event together with another non-differentially expressed gene (Supplementary Figure 10A). The proteins encoded by these genes contained the motif of the Membrane-Associated Proteins in Eicosanoid and Glutathione metabolism superfamily (MAPEG, PFAM01124) and residues that bind glutathione, supporting the inferred annotation (Supplementary Figure 10B). Another GT acting on lipids was up-regulated at high salt (m.14632, 7.7-fold increase). It was related to the peroxiredoxin 6 (Prdx 6) family and possessed the conserved motif PVCTTE and the putative catalytic triad His^39^-Cys^47^-Arg^132^ which confers peroxidase activity (Supplementary Figure 11; Choi et al., 1998; Nevalainen, 2010).

One strongly up-regulated gene related to the Beta class of GTs (ORF m.57692, 9.1-fold increase, 1,280 TPM at 30% salt, rank 136) was closely related to another non-differentially expressed gene (ORF m.3188), suggesting gene duplication and neo-functionalization (Supplementary Figure 12A). The proteins encoded by these two genes displayed the N-terminal and C-terminal domains of Beta GTs (CD03057 and CD03188, respectively) and contained residues implicated in binding glutathione as well as crucial residues strictly conserved in Beta class GTs (Supplementary Figure 12B; Casalone et al., 1998; Allocati et al., 2000; Inoue et al., 2000; Federici et al., 2007, 2009). Another gene coding for a protein related to cytosolic GT was also up-regulated (m.69131, 3.8-fold increase, Table 3) and encoded the N-terminal domain CD00570 and C-terminal domain CD10292.

**Figure 8:**
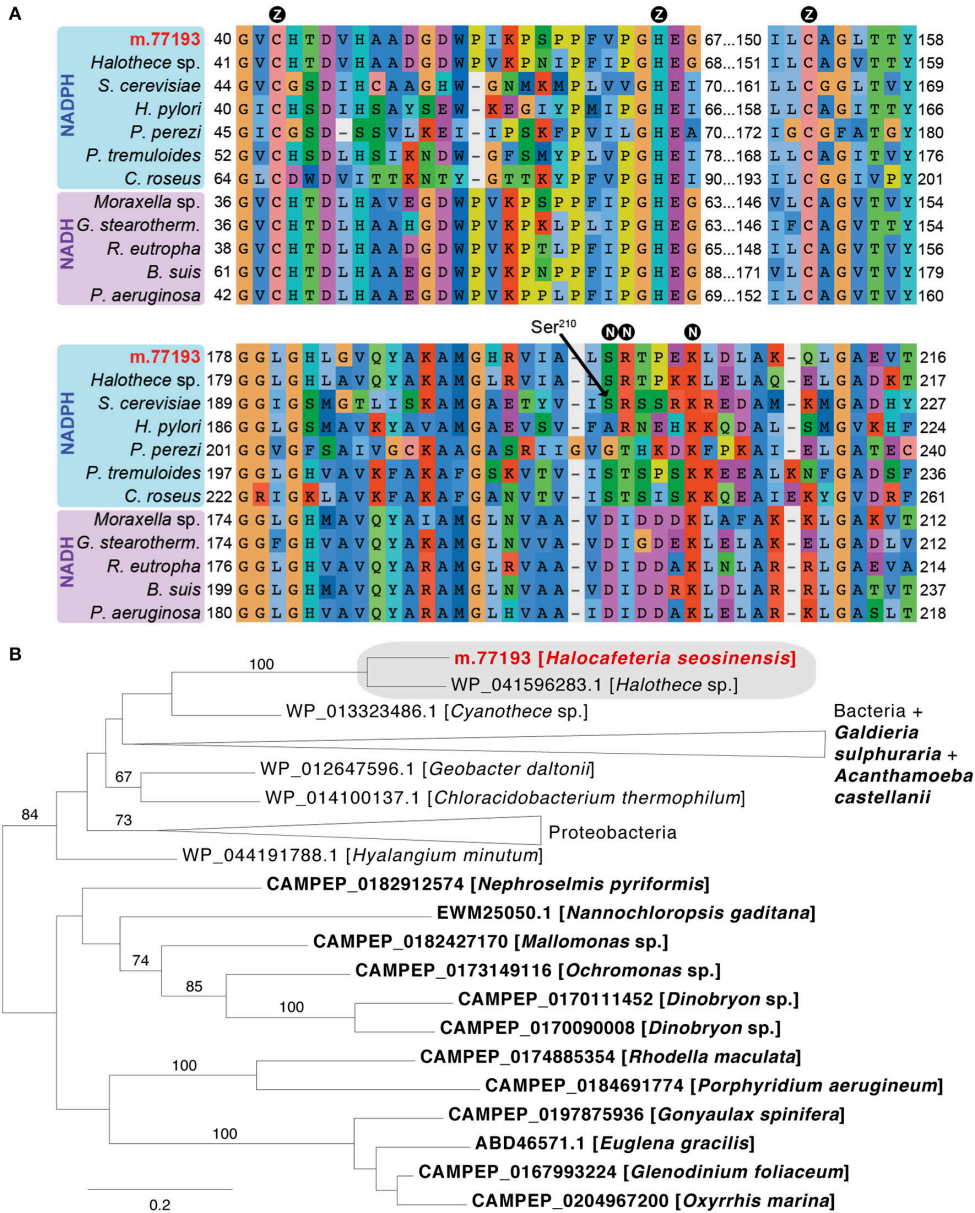
**(A)** Partial alignment of zinc-dependent alcohol dehydrogenase sequences showing conservation of residues binding the catalytic zinc ion (circled Z) and NAD(P)H (circled N). At position 210 (Ser^210^, *Saccharomyces cerevisiae* 1Q1N numbering), small neutral amino acids (Ala, Gly, Ser) confer specificity for NADPH, while NADH-dependent enzymes instead have a negatively charged residue. In NADPH-dependent dehydrogenase, the following position contains a positively charged residue (as in the *S. cerevisiae* enzyme) or a threonine (as in the *Pelophylax perezi* enzyme 1P0C; Rosell et al., 2003) that interacts with the NADPH terminal phosphate group. The alignment contains the *H. seosinensis* sequence (in red), its closest sequence in the NR database (*Halothece* sp. WP_041596283.1), and sequences for characterized NADPH-dependent enzymes from *S. cerevisiae* (1Q1N), *P. perezi* (1P0C), *Helicobacter pylori* (3TWO), *Populus tremuloides* (1YQD) and *Catharanthus roseus* (5H81), and for NADH-dependent enzymes from *Moraxella* sp. (4Z6K), *Geobacillus stearothermophilus* (3PII), *Ralstonia eutropha* (3S1L), *Brucella suis* (3MEQ), and *Pseudomonas aeruginosa* (1LLU). **(B)** Maximum-likelihood phylogenetic tree for zinc-dependent alcohol dehydrogenase showing strongly supported clustering (gray box) of sequences from *H. seosinensis* (in red) and the cyanobacterium *Halothece* sp. to the exclusion of all other bacterial and eukaryotic sequences (eukaryotes in bold). Bootstrap values (>50%) are indicated on branches. The scale bar indicates the expected substitutions/site.

One gene coding for a protein related to cinnamyl-alcohol dehydrogenase (CD08297) was 202-fold overexpressed (m.77193, from 2 TPM at 15% salt to 627 TPM at 30% salt, PPDE = 1.0, DESeq2 adjusted *p* = 7.8 × 10^−156^, voom-limma adjusted *p* = 8.2 × 10^−5^). The protein was predicted to be a NADPH-dependent zinc-binding alcohol dehydrogenase, based on the identification of conserved residues (Figure 8A). This enzyme could be involved in regeneration of NADPH, which, as the cofactor for enzymes such as glutathione reductase and thioredoxin reductase, provides the reducing power required to quench ROS (Marty et al., 2009). Phylogenetic analysis grouped this sequence with a zinc-dependent alcohol dehydrogenase sequence from the halotolerant cyanobacterium *Halothece* sp. PCC 7418 (Garcia-Pichel et al., 1998) with maximum support (Figure 8B). This gene contained an intron in the 5′-UTR (Supplementary Figure 9B) and it had high transcript abundance, showing that it was not a bacterial contamination; it is a strong candidate for origin via LGT.

In addition to up-regulating genes for directly neutralizing ROS or removing electrophilic groups from proteins, lipids and metabolites, *H. seosinensis* also overexpressed several chaperones that repair misfolded proteins, or minimize their detrimental impact (Table 4). For example, all genes encoding the alpha-crystallin domain of the small heat shock proteins (sHSPs, PFAM00011) were differentially expressed, with three being 3.8- to 6.7-fold up-regulated and one being 10-fold repressed. Another chaperone involved in protein disaggregation, the ATP-dependent chaperone ClpB, was also up-regulated at high salt (m.91451, 6.3-fold increase). Furthermore, *H. seosinensis* expressed eight genes encoding the Hsp70 domain (PFAM00012) with one coding for a cytosolic Hsp70 being very strongly up-regulated at high salt (m.81151, 15.2-fold increase). Average transcript abundance for this gene was 1,657 TPM at 30% salt, corresponding to the 110th most expressed transcript.

**Table 4:** **Differentially expressed genes coding for chaperones in *Halocafeteria seosinensis***.

**ORF names**	**Abundance (TPM)**		**EBSeq**		**DESeq2**		**VOOM-LIMMA**	
	**15% salt**	**30% salt**	**PPDE**	**Post fold change**	**Adjusted *p*-values**	**log_2_FC**	**Adjusted *p*-values**	**log_2_FC**
**SMALL HEAT SHOCK PROTEINS**								
m.64653	91.22	816.55	1.00	6.71	NA	2.54	0.010	2.89
m.65830	163.10	908.44	1.00	4.24	9.2E-06	1.98	0.009	2.22
m.67185	54.77	275.20	1.00	3.75	0.00024	1.80	0.013	2.13
m.8880	20.89	2.88	0.99	0.10	7.7E-09	−3.04	0.018	−2.98
**ATP-DEPENDENT ClpB PROTEASE**								
m.91451	12.06	100.83	1.00	6.27	1.2E-10	2.54	0.003	2.74
**HSP70-LIKE PROTEIN**								
m.81151	83.29	1664.31	1.00	15.25	4.4E-20	3.75	0.001	3.99

*TPM, averaged transcripts per million; PPDE, Probability of being Differentially Expressed; Post Fold Change, posterior fold change (30% over 15% salt); log_2_FC, log_2_ fold change (30% over 15% salt); NA, not available due to an extreme count outlier in one of the samples*.

In oxidative stress conditions, quinones are vulnerable to one-electron reduction that generates reactive semiquinones (Wrobel et al., 2002). *H. seosinensis* expressed three genes encoding the NAD(P)H: quinone oxidoreductase (NQO) type IV domain (TIGR01755), which can prevent semiquinone formation. These were all up-regulated at high salt (2.1- to 12-fold increase) and encoded conserved functional residues that supported the inferred annotation (Supplementary Figure 13A). Furthermore, one of them (m.35863) had very high transcript abundance at 30% salt (average of 1,581 TPM, rank 118). Phylogenetic analysis indicated that these genes probably arose through duplications after divergence from *Cafeteria roenbergensis* (Supplementary Figure 13B).

### Lipid metabolism

Membrane adaptation to salinity is a complex phenomenon and transcriptomic analyses can only provide a very partial picture. Nonetheless, investigation of the *H. seosinensis* transcriptional program strongly suggested that lipid metabolism was affected, especially synthesis and transport of sterol, phosphatidylinositol and phosphatidylethanolamine, and regulation of the length of phospholipids.

Several genes involved in sterol synthesis were repressed at high salt, while none showed statistically supported increases in expression (Table 5). The repressed genes coded for proteins related to cycloeucalenol isomerase (m.88947, 2.7-fold repression), squalene monooxygenase (m.88587, 5.3-fold repression) and sterol 24-C-methyltransferase (m.73826, 2.5-fold repression). Consistently, genes involved in sterol transport were also repressed at high salt. Five proteins affiliated to the Niemann-Pick type C1 (NPC1) protein (TIGR00917) were expressed, and among them, four were repressed at high salt (4.2- to 220-fold repression; NPC1-related proteins in Table 5). These proteins all encoded at least one sterol-sensing domain (PFAM12349) that potentially monitors the free sterol level in the membrane (Li et al., 2016). Since NPC1 proteins might also be involved in the transport of sphingolipids (Malathi et al., 2004; Lloyd-Evans and Platt, 2010; Feldman et al., 2015), repression of these genes in *H. seosinensis* indicated that transport of lipids, sterol or sphingolipid, was affected.

**Table 5:** **Differentially expressed genes involved in lipid metabolism in *Halocafeteria seosinensis***.

**ORF names**	**Abundance (TPM)**		**EBSeq**		**DESeq2**		**VOOM-LIMMA**		**Annotation**
	**15% salt**	**30% salt**	**PPDE**	**Post fold change**	**Adjusted *p*-value**	**log_2_FC**	**Adjusted *p*-value**	**log_2_FC**	
**STEROL SYNTHESIS AND TRANSPORT**									
m.88587	136.85	34.23	1.00	0.19	2.3E-17	−2.35	0.0011	−2.46	squalene monooxygenase
m.73826	264.89	138.27	1.00	0.40	1.8E-13	−1.31	0.0021	−1.30	sterol 24-C-methyltransferase
m.88947	29.67	14.34	1.00	0.37	1.3E-13	−1.43	0.0019	−1.44	cycloeucanelol cycloisomerase
m.26733	19.08	0.10	1.00	0.0045	3.5E-83	−7.53	0.0004	−7.78	NPC1-related proteins
m.35144	13.30	1.88	1.00	0.11	6.7E-29	−3.12	0.0005	−3.21	NPC1-related proteins
m.88487	33.37	8.27	1.00	0.19	8.7E-22	−2.32	0.0008	−2.32	NPC1-related proteins
m.41605	32.76	10.53	1.00	0.24	6.0E-18	−2.02	0.0011	−2.04	NPC1-related proteins
m.17881	5.03	39.01	1.00	5.88	5.1E-31	2.53	0.0003	2.61	sterol O-acyltransferase
**PHOSPHATIDYLINOSITOL (PI) AND PHOSPHATIDYLETHANOLAMINE SYNTHESIS**									
m.10411	144.51	60.42	1.00	0.32	2.1E-09	−1.63	0.0042	−1.59	PI synthase
m.47108	82.72	53.99	1.00	0.50	1.0E-05	−0.99	0.0103	−0.98	phosphoethanolamine cytidylyltransferase
**FATTY ACID DESATURASES (FAD)**									
m.39033	197.84	116.91	1.00	0.45	5.6E-23	−1.14	0.0014	−1.12	delta12 FAD
**FATTY ACID ELONGASES (FAE)**									
m.59689	85.48	52.70	1.00	0.48	2.9E-15	−1.05	0.0021	−1.03	long-chain FAE
m.60210	170.73	92.75	1.00	0.41	7.9E-15	−1.26	0.0018	−1.25	long-chain FAE
m.45555	71.28	36.35	0.97	0.38	1.0E-05	−1.34	0.0133	−1.30	long-chain FAE

*TPM, averaged transcripts per million; PPDE, Probability of being Differentially Expressed; Post Fold Change, posterior fold change (30% over 15% salt); log_2_FC, log_2_ fold change (30% over 15% salt)*.

In addition to transcriptional regulation, sterol homeostasis in *H. seosinensis* seemed to be achieved by cycles of esterification and hydrolysis. This represents the main short-term sterol regulation mechanism in mammals, yeasts and plants, and was also described in the apicomplexan parasite *Toxoplasma gondii* (Yang et al., 1996; Schaller, 2004; Lige et al., 2013; Rogers et al., 2015). *H. seosinensis* expressed two genes related to sterol O-acyltransferase (SOAT; ORFs m.17881 and m.78053) which leads to withdrawal of sterol from the membrane and to accumulation of sterol esters in cytoplasmic fat droplets (Rogers et al., 2015). The up-regulation at high salt of m.17881 (5.9-fold increase; Table 5) suggests another mechanism by which sterol abundance in the membrane may be lowered in this condition (in addition to the lowered sterol production suggested above). Conversely, hydrolysis by sterol esterases results in free sterols being inserted back in the membrane, however the genes potentially coding for this enzyme were not differentially expressed in *H. seosinensis*.

**Figure 9:**
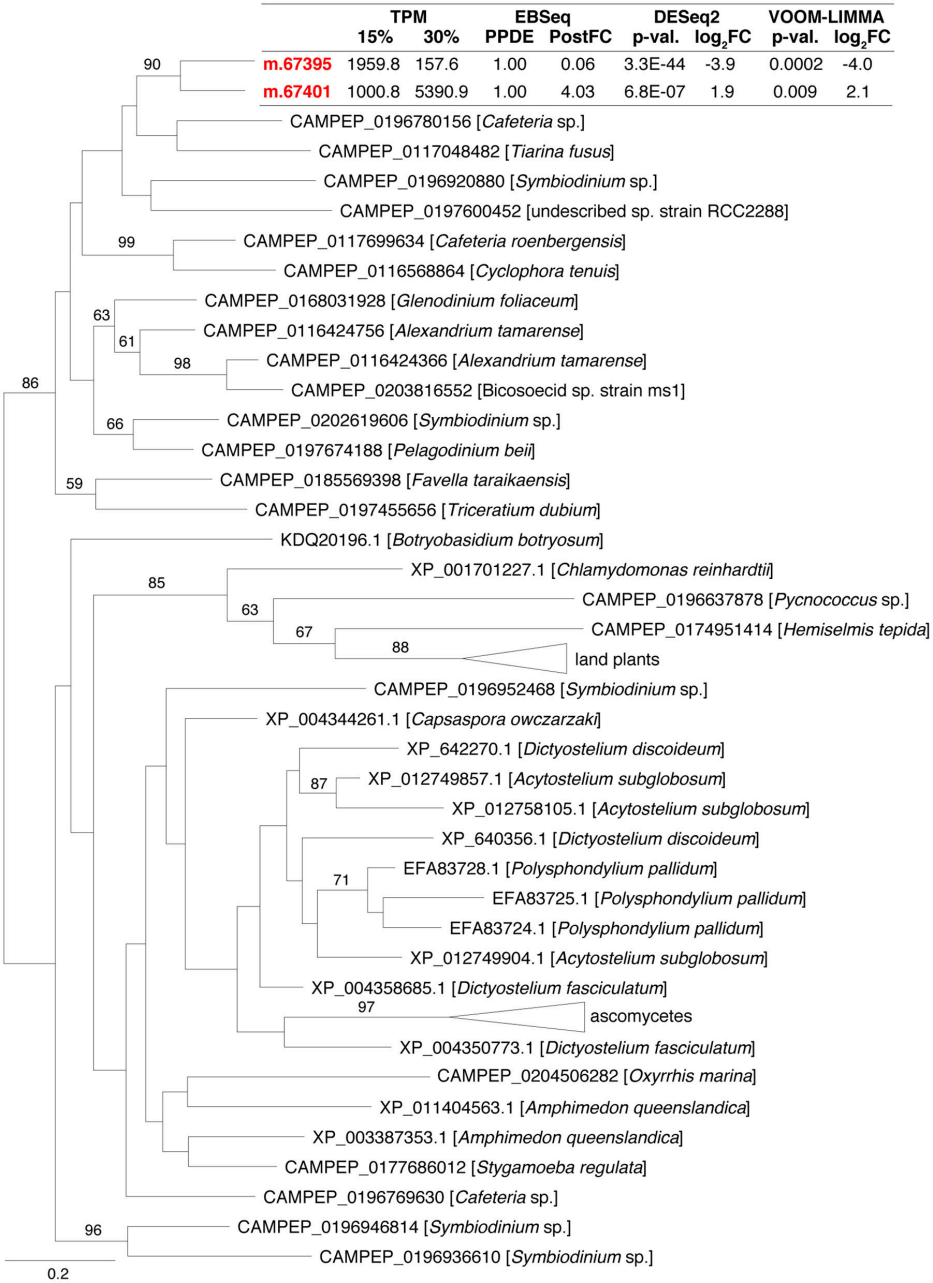
**Maximum-likelihood phylogenetic tree for gene duplication cluster encoding phosphatidylglycerol/phosphatidylinositol transfer proteins**. Bootstrap values (>50%) are indicated on branches. The scale bar indicates the expected substitutions/site. For *H. seosinensis* sequences (in bold), expression values are indicated: TPM, averaged transcript per million at 15 or 30% salt; PPDE, Posterior Probability of being Differentially Expressed; PostFC, Posterior Fold Change calculated by EBSeq; *p*-val., adjusted *p*-value; log_2_FC, log_2_ Fold Change calculated either by DESeq2 or voom-limma.

A substantial difference in expression pattern between two closely related genes that were affiliated to phosphatidylglycerol/phosphatidylinositol transfer proteins (PG/PI-TP), suggested that membrane phospholipids are adjusted in response to increased salinity. These two genes each encoded a MD-2-related lipid recognition domain (PFAM02221), and were identified as being recently duplicated (i.e., after the divergence of *C. roenbergensis* and *H. seosinensis* from their common ancestor, Figure 9). They had drastically different expression patterns, with m.67395 being 16-fold repressed and m.67401 4.0-fold up-regulated in high salt. In addition, both had extremely high transcript abundances in their respective favored salinity (1,959 TPM, rank 95 at 15% salt, and 5,366 TPM, rank 7 at 30% salt, respectively), as was the case of the homologous transcript in *C. roenbergensis* (MMETSP coding sequence MMETSP0942-20121206|242_1; 4,317 TPM, rank 32), suggesting these proteins have important functions. At least one of the *H. seosinensis* proteins (m.67401) potentially functions at the plasma membrane, since it has a signal peptide.

Genes predicted to be involved in phospholipid synthesis in *H. seosinensis* were not differentially expressed, with the exception of two enzymes: phosphatidylinositol synthase (PIS; m.10411, 3.1-fold repression) and phosphoethanolamine cytidyltransferase (PEC; m.47108, 2.0-fold repression), involved in phosphatidylinositol and phosphatidylethanolamine synthesis, respectively (Table 5). The physiological consequence of repression of phosphatidylinositol synthesis remains uncertain since phosphatidylinositol is also the precursor for molecules involved in a diverse array of biological functions (e.g., Divecha and Irvine, 1995; Martin, 2001; Sun et al., 2007; Paulick and Bertozzi, 2008). In contrast, phosphatidylethanolamine is a major constituent of the eukaryotic plasma membrane (Vance and Tasseva, 2013). PEC is highly specific for its substrate phosphoethanolamine in both mammals and plants and, as the rate-limiting step of the pathway, is considered the key-regulatory enzyme of phosphatidylethanolamine synthesis (Sundler and Akesson, 1975; Wang and Moore, 1991; Vermeulen et al., 1994; Bladergroen and van Golde, 1997; Tang and Moore, 1997; Maheshwari et al., 2013). This result relates to salt adaptation in bacterial membranes, where phosphatidylethanolamine is predicted to destabilize the bilayer phase at higher salinities (Russell, 1989).

The length and saturation level of fatty acyl chains both impact membrane fluidity, with shorter chain lengths and a greater number of double bonds increasing fluidity (Lodish et al., 2000; Beney and Gervais, 2001). A set of genes encoding long (>20 carbons; Oh et al., 1997) chain fatty acid elongases (PFAM01151) were repressed at high salt (2.1- to 2.6-fold), concordant with a theoretical need for shorter acyl chains in this condition (Table 5). Genes for several putative desaturases (with domain PFAM00487) were identified, but only one was differentially expressed. This was a gene related to delta12 fatty acid desaturases (CD03507) that was actually 2.2-fold repressed at high salt (m.39033; Table 5). If this enzyme indeed acts on lipid exported to the plasma membrane, its down-regulation at high salt is unexpected since it would theoretically lead to lower membrane fluidity in a condition that causes reduced fluidity.

### Carbohydrate and amino acid metabolism

A substantial proportion of the genes related to glycoside hydrolases and sugar transporters were up-regulated at high salt. More specifically, six of them (showing 2.1- to 9.1-fold increases) were related to hexaminidase, polygalacturonase, α-xylosidase, and α-galactosidase (glycoside hydrolase families 20, 28, 31, and 36), and five genes (showing 3.1- to 44-fold increases) were related to transporters of the Major Facilitator Superfamily (domains of transporters for sugar—PFAM00083, for nucleotide-sugar—COG5070, and for triose-phosphate—PFAM03151; Table 6). Enhanced expression of these genes indicated that the intracellular carbohydrate content might be higher at high salt. In line with this possibility was the up-regulation (2.4-fold) of fructosamine-3-kinase (PFAM03881). This enzyme removes unwanted fructosamine residues added to proteins during spontaneous glycation, a phenomenon that is proportional to intracellular glucose concentration (Delpierre and Van Schaftingen, 2003). This enzyme in *H. seosinensis* possessed the conserved aminoglycoside kinase motif PxLXHGDLWSxN (from amino acid positions 199–210 in ORF m.82266).

**Table 6:** **Expression of genes involved in carbohydrate metabolism in *Halocafeteria seosinensis***.

**ORF names**	**Abundance (TPM)**		**EBSeq**		**DESeq2**		**VOOM-LIMMA**	
	**15% salt**	**30% salt**	**PPDE**	**Post fold change**	**Adjusted *p*-value**	**log_2_FC**	**Adjusted *p*-value**	**log_2_FC**
**GLYCOSYL HYDROLASES**								
m.38126	0.22	2.70	1.00	8.83	1.0E-14	3.09	0.001	3.36
m.17615	1.66	8.05	1.00	4.02	1.8E-04	1.89	0.010	2.05
m.13232	1.60	7.94	1.00	3.97	5.0E-14	1.96	0.002	2.01
m.67061	12.86	39.87	1.00	2.46	3.9E-10	1.29	0.003	1.32
m.46314	20.08	57.80	1.00	2.30	3.3E-04	1.17	0.016	1.25
m.78119	14.78	38.53	1.00	2.07	0.0011	1.03	0.024	1.05
**SUGAR TRANSPORTERS**								
m.89759	1.17	69.25	1.00	44.08	7.9E-112	5.41	8.2E-05	5.56
m.27262	4.84	61.80	1.00	9.40	1.2E-26	3.17	3.7E-04	3.41
m.51724	1.85	10.65	1.00	4.94	NA	2.08	0.018	2.42
m.52982	1.70	7.40	1.00	3.47	1.4E-10	1.76	0.002	1.78
m.15888	6.44	25.46	1.00	3.10	1.7E-12	1.61	0.002	1.62
**FRUCTOSAMINE-3-KINASE**								
m.82266	61.77	195.44	1.00	2.44	5.61E-05	1.26	0.015	1.28
**PROTEIN GLYCOSYLATION**								
m.59896	0.07	5.36	1.00	56.61	7.97E-23	5.38	0.001	5.88
m.15355	9.55	28.21	1.00	2.38	6.22E-07	1.23	0.005	1.28

*TPM, averaged transcripts per million; PPDE, Probability of being Differentially Expressed; Post Fold Change, posterior fold change (30% over 15% salt); log_2_FC, log_2_ fold change (30% over 15% salt); NA, not available due to an extreme count outlier in one of the samples*.

Hypothetically, increased expression of carbohydrate-related enzymes and transporters could be linked to protein glycosylation, potentially leading to increased protein solubility in conditions with lower water activity (Schülke and Schmid, 1988; Tams and Welinder, 1995; Tams et al., 1999). Concordantly, among the five genes encoding the domain of the glycosyl transferase family 41 (PFAM13844, *O*-linked β-*N*-acetylglucosamine transferases), two were 2.2- and 57-fold up-regulated, suggesting that protein glycosylation was stimulated at high salt (Table 6).

The class “Amino acid transport and metabolism” was enriched in duplicated genes (adjusted *p* = 6.31 × 10^−6^, Figure 3). Forty percent (12/30) of duplicated genes assigned to this class encoded amino acid transporters and peptidases (e.g., Supplementary Figures 14, 15). Amino acids and derivatives are common osmolytes (Galinski, 1995), thus diversification of genes involved in acquisition of amino acids through import or following protein catabolism could be linked to salt adaptation. Although the potential role of amino acids and derivatives in maintaining osmotic equilibrium in *H. seosinensis* was discussed previously (Harding et al., 2016), further experimental work is required to test this hypothesis.

## Discussion

Biological reactions can be influenced by a myriad of mechanisms like allosteric enzymatic regulation, stability of mRNA or proteins involved, and enzymatic processivity. In this context, transcriptomic analyses provide a partial picture of the cellular responses to different environments. Nonetheless, our analysis identified a number of plausible contributors to *H. seosinensis* high-salt resistance including enzymes and pathways that act in ion homeostasis, signal transduction, and stress control, as well as in lipid, carbohydrate and amino acid metabolism. These represent candidates for involvement in molecular adaptations to high salt in *H. seosinensis* that can be pursued experimentally in the future.

### Signaling and the stress response induced by high salt

Expression of several genes coding for proteins involved in signal transduction was highly up-regulated at high salt. Based on sequence analyses, the nature of the stimuli that initiated these cascades remained elusive, especially since sensing domains are commonly very divergent, as for GPCR (Oliveira et al., 1999) or histidine kinases (Stock et al., 2000; Anantharaman et al., 2001; Aravind et al., 2002), or were not detected in *H. seosinensis*, possibly because the membrane itself could trigger activity, as for some guanylate cyclases (Reddy et al., 1995; Cooper et al., 1998). Some of these genes are known to function in drought and osmotic change signaling; examples include CHASE domain-containing enzymes such as the histidine kinases AHK2 and AHK3 in plants (Tran et al., 2007) and adenylyl cyclase G in *Dictyostelium* (van Es et al., 1996). Based on their expression profile in *H. seosinensis*, these genes might be important in long-term salt adaptation.

It is well known that environmental stresses lead to increased cellular levels of ROS (Lushchak, 2011; Sharma et al., 2012). The ability to survive such stress resides in the capacity to manage these destructive ions that otherwise react with DNA, proteins and lipids (Yu, 1994). For example, plant species with greater antioxidant capacities show a greater resistance to salt stress (Panda and Das, 2005). Expression of genes involved in ROS detoxification, several of which, like SOD and peroxidase, were very abundantly transcribed at high salt by *H. seosinensis*, suggested that ROS level was higher at high salt and that this ROS detoxification ability likely contributes to the organism's tolerance of hypersaline conditions. These included genes coding for proteins related to enzymes linked to increased resistance to oxidative stress, for example those of the alkylhydroperoxidase family (Hillas et al., 2000; Paterson et al., 2006) and Beta class glutathione transferases (Favaloro et al., 2000; Allocati et al., 2003; Tamburro et al., 2004).

Furthermore, several transcription factors linked to stress response in other organisms were up-regulated at high salt in *H. seosinensis*. These included ATF2, a BZIP-domain containing factor to which m.26350 was related (40-fold increase, Supplementary Table 4). This factor is activated when phosphorylated by stress-activated protein kinases in response to varying stimuli like DNA damage or ROS levels (van Dam et al., 1995). Other transcription factors potentially related to stress response and up-regulated at high salt include (i) sirtuins that are implicated in a wide range of cellular processes including tolerance to oxidative stress (Feige and Auwerx, 2008), (ii) transcription factors of the MYB superfamily that regulate abiotic stress response gene expression in plants (Baldoni et al., 2015; Roy, 2016), and (iii) AP2 domain-containing factors that are involved in environmental stress response pathways (Licausi et al., 2013; Dey and Vlot, 2015). In addition, the up-regulation of heat shock factors, which regulate the expression of heat shock proteins, also indicated that higher stress levels affected gene expression in *H. seosinensis* grown at high salt.

Concordantly, several highly salt-responsive genes coded for chaperones, for example Hsp70 domain-containing proteins, sHSPs that bind to denatured proteins to prevent irreversible aggregation (Ehrnsperger et al., 1997; Lee et al., 1997; Lee and Vierling, 2000), and ClpB, which dynamically mediates the disaggregation of stress-damaged proteins (Hodson et al., 2012). The ClpB chaperone is particularly important since, whereas the Hsp70 system can independently correct populations of small aggregates, resolubilisation of large aggregates requires ClpB (Goloubinoff et al., 1999; Diamant et al., 2000). Up-regulation of these genes indicated that the protein pool was under higher threat at high salt. The over-representation of transcripts of glutaredoxin, which reduces protein disulfides and glutathione-protein mixed disulfides (Lillig et al., 2008), was also consistent with this interpretation. Cysteinyl residues are particularly vulnerable to ROS since they are amongst the most easily oxidized residues in proteins (Lii et al., 1994; Ravichandran et al., 1994). In *S. cerevisiae*, mutants of glutaredoxins Grx1 and Grx2 are more susceptible to oxidants like hydroperoxides, paraquat and iron chloride, and while overexpression of these genes improves tolerance to oxidants (Luikenhuis et al., 1998; Collinson et al., 2002; Collinson and Grant, 2003).

Overexpression of lipid-specific detoxification enzymes suggested that phospholipids were also threatened by oxidants in *H. seosinensis*. Overexpressed enzymes included one related to MGT3 (encoded by ORF m.21576) which displays wide-specificity glutathione transferase activity toward lipophilic substrates (Jakobsson et al., 1997; Chen et al., 2011) and one related to Prdx 6 (m.14632), which reduces phospholipid hydroperoxides (Fisher, 2011). Interestingly, Prdx 6 was previously shown to be transcriptionally regulated during oxidative stress in human systems and *Plasmodium yoelii* (Kawazu et al., 2003; Kim et al., 2003; Chowdhury et al., 2009; O'Flaherty and de Souza, 2011). In summary, ROS were most probably major triggering factors of the high-salt stress response in *H. seosinensis*, and overexpression of a battery of anti-oxidant genes likely contributed to increased *H. seosinensis* stress resistance.

### Lipid metabolism and a potential need for increased membrane fluidity at high salt

As a response to variations in salinity, eukaryotic organisms adapt their membrane lipid composition by adjusting the relative proportion of phospholipid head groups and sterols, where higher amounts of the latter decrease membrane fluidity by reducing lipid acyl chain mobility (Demel and De Kruyff, 1976; Quinn, 1981). Consistently, an increase in salinity induced the down-regulation of genes involved in sterol synthesis and transport in *H. seosinensis*. In addition, cycles of esterification potentially regulated sterol content of the membrane, where higher levels of SOAT transcripts are predicted to imply increased sterol withdrawal from the membrane. In *Arabidopsis thaliana*, overexpression of sterol acyltransferase 1 was shown to lead to a 2-fold increase of sterol ester and a reduction from 59% of free sterol in wild type individuals to 28% in transgenic plants (Chen et al., 2007). Thus, over-expression of these genes in *H. seosinensis* could be linked to membrane sterol homeostasis where less membrane sterol would be required to ensure a fluid membrane at high salt. Interestingly, a decrease in membrane sterol content at increasing salinity was measured directly in the halotolerant yeasts *Yarrowia lipolitica* and *Debaryomyces hansenii* (Tunblad-Johansson et al., 1987; Andreishcheva et al., 1999).

Twelve molecules of oxygen are required to synthesize one sterol molecule (Summons et al., 2006). As mentioned previously, oxygen solubility is reduced at high salt, raising the question of whether the repression of sterol synthesis at high salt was actually a result of lower oxygen availability. However, this is unlikely since, although yeasts are auxotrophic for sterol when grown in complete absence of oxygen (Andreasen and Stier, 1953), low oxygen availability actually stimulates transcription of sterol biosynthetic enzymes (Hughes et al., 2005; Todd et al., 2006; Chun et al., 2007; Synnott et al., 2010). Meanwhile, the transcriptome of *H. seosinensis* indicates that it is respiring at high salt, implying some oxygen availability. This argues against an oxygen-dependent repression of sterol synthesis at high salt in *H. seosinensis* and suggests that another factor was involved, a need for increased membrane fluidity being the most plausible possibility.

Another indication of a need for increased membrane fluidity at high salt was the repression of phosphatidylethanolamine synthesis, as previously observed in the halotolerant yeast *D. hansenii*, the halophilic yeast *Phaeotheca triangularis*, and in halotolerant and halophilic bacteria (Vreeland et al., 1984; Russell, 1989, 1993; Andreishcheva et al., 1999; Turk et al., 2004). Extracellular salinity affects biological membranes by favoring transition from the bilayer (lamellar) phase to the hexagonal-II (non-bilayer) phase of certain lipids (Beney and Gervais, 2001; Simonin et al., 2008). The down-regulation of synthesis of phosphatidylethanolamine, which is relatively prone to adopting a hexagonal-II phase at higher salinities (Harlos and Eibl, 1981; Sutton et al., 1990), could contribute to avoiding the formation of microdomains of hexagonal-II phase lipids, and thus prevent alterations of the membrane permeability (Russell, 1989).

Reminiscent of an alteration of the membrane lipid composition was the strongly contrasting expression patterns of two genes (m.67395, 16-fold repression and m.67401 with 4.0-fold up-regulation) that originated from a recent duplication and are affiliated with a group of phosphatidylglycerol/phosphatidylinositol transfer proteins that are responsible for the intermembrane movement of phospholipids (Wirtz, 1991). The closest characterized homolog available, expressed by *Aspergillus oryzae*, was shown to preferentially transfer PG and PI but also phosphatidylcholine, phosphatidylethanolamine and phosphatidylserine (Record et al., 1995). Transcription of this gene in *A. oryzae* was stimulated by phospholipid supplementation of the medium, and co-accumulation of mRNA transcripts and the protein was observed (Record et al., 1999). Further experiments are required to determine what types of phospholipid are transferred by both proteins as well as their cellular localization in *H. seosinensis*. Nonetheless, this observation is consonant with phospholipid composition varying as a function of salinity.

### Stimulation of carbohydrate metabolism at high salt

Our results suggest that carbohydrate metabolism was stimulated in the high salt condition, possibly resulting in the accumulation of osmolytes (though see below) and/or in increased protein glycosylation. The latter is suggested by the salt-dependent overexpression of *O*-linked β-*N*-acetylglucosamine transferases in *H. seosinensis. O*-linked glycosylation can have myriad effects on proteins. For example, it can affect protein structure by increasing stability, can regulate enzymatic activity, and can modulate proteolytic cleavage that influences protein expression and processing (Van den Steen et al., 1998). However, in response to exposure to a variety of stressors (salt, hydrogen peroxide, heat, UV light, heavy metals), *O*-linked glycosylation increases on a large number of proteins as a protective mechanism, at least in metazoan cells (Zachara et al., 2004; Selvan et al., 2015). The protective effect of *O*-linked glycosylation is partly explained by modulation of HSP70 and HSP40 expression and persistence (Zachara et al., 2004). It is possible that the overexpression of *O*-linked β-*N*-acetylglucosamine transferases in *H. seosinensis* is related to a similar protective role.

Metabolized carbohydrates are commonly used as organic osmolytes. However, since the reactive reducing end of sugars could threaten other cellular components, osmoprotective carbohydrates are typically non-reducing saccharides, like trehalose, or they are modified by addition of a small neutral molecule, like glycerol, glyceramide, or glyceric acid (Roberts, 2005). We could not unambiguously identify enzymes involved in the synthesis or transport of such carbohydrates in *H. seosinensis*. Investigation of the intracellular metabolites by H-NMR, HPLC, or mass spectrometry and of the glycosylated protein pool, for example by lectin purification followed by a labeling procedure, will be required to clarify the role of carbohydrates.

### Evolution by gene duplication and lateral transfer

We recorded several cases where salt-responsive genes were involved in duplication events, indicating that gene duplication might have created genetic novelties favoring *H. seosinensis* adaptation to high salt, possibly through acquisition of different substrate specificity, subcellular localization or multimeric states. For example, the *H. seosinensis* genome encoded 13 extremely differentially expressed genes coding for P2XR. The human genome encodes seven P2X proteins that assemble into homo- or hetero-trimeric receptors, multiple combinations allowing for functional versatility (North, 2002). This P2XR multimeric state was also observed in *D. discoideum*, whose genome encodes five *P2X* genes with the corresponding proteins all localized to the contractile vacuole, an organelle involved in osmoregulation (Aravind et al., 2002; Fountain et al., 2007; Ludlow et al., 2009). Disruption of the *p2xA* gene in *D. discoideum* strain AX4 resulted in an inability to regulate cell volume in hypotonic solution (Fountain et al., 2007), although this was not observed when the gene was disrupted in a different strain, AX2 (Ludlow et al., 2009; Sivaramakrishnan and Fountain, 2013). Our analysis revealed that a high copy number for *P2X* genes is rather rare in other genomes, suggesting that these patterns of differential gene expression and gene duplication in *H. seosinensis* may have a role in salt adaptation. We speculate that multiple subunit combinations could lead to fine-tuned environmental stress responses.

In halophilic yeasts, expansions of gene families encoding cation transporters and P-type ATPases were observed, perhaps allowing a greater potential for adaptation to varying salt conditions (Lenassi et al., 2013; Zajc et al., 2013). A significant enrichment of ion transporter genes was also detected in *H. seosinensis*. However, an enrichment in duplicated genes assigned to this class was also detected in the genomes of *N. gaditana* and *G. theta* (adjusted *p*-values of 0.041 and 3.9 × 10^−12^, respectively), implying that this situation is not unique to extreme halophiles (Figure 3). Nonetheless, duplication of ion transporter genes might be a requirement for salt adaptation.

In prokaryotes, LGT is accepted as another important mechanism that has the potential to increase the fitness of the recipient cell (Battistuzzi and Brown, 2015). Although the importance of LGT to eukaryotic evolution is still debated (Ku et al., 2015), its incidence in microbial eukaryotes has been increasingly documented (Keeling and Palmer, 2008; Andersson, 2009; Soucy et al., 2015). Several cases have been reported where LGT was inferred to be a probable driver of niche adaptation, including adaptation to anaerobic and parasitic lifestyles, and to rumen, sea ice, and soil habitats (Richards et al., 2003; Eichinger et al., 2005; Ricard et al., 2006; Raymond and Kim, 2012; Stairs et al., 2014; Xu et al., 2016; Eme et al., 2017). Furthermore, in the polyextremophile red alga *G. sulphuraria*, adaptation to extreme environments was likely facilitated by lateral acquisition of genes coding for ion transporters, osmolyte synthesizers, and toxic metal pumps and neutralizers (Schönknecht et al., 2013).

Although we did not search comprehensively for LGT candidates in *H. seosinensis*, we did identify two cases of abundantly transcribed and strongly up-regulated genes that were most probably acquired horizontally from bacteria: a peroxidase that was the 11th most expressed transcript at high salt and a NADPH-dependent alcohol dehydrogenase. Interestingly, the closest available sequences to these genes belonged to extremophilic bacteria or to bacteria that were isolated from extreme environments, or survive high levels of radioactivity. The closest NADPH-dependent alcohol dehydrogenase sequence was from *Halothece* sp. PCC 7418, an isolate from saline Solar Lake (Garlick et al., 1977). The *Halothece* and *Halocafeteria* protein sequences were 79% identical, thus arguing against the “70% rule” of Ku and Martin (2016) that considers eukaryotic sequences >70% identical to prokaryotic sequences as originating from contamination. We are confident this is not the case here since the corresponding gene actually contained a spliceosomal intron, as well as the transcript abundance being >50-fold higher (at high salt) than any known contaminant (see Section Evaluation of Prokaryotic Contamination Based on Transcript Abundance). Moreover, no other cyanobacterial sequences were detected in our data. The sequences that were closest to *H. seosinensis* peroxidase were from bacteria like the thermophile *Deferrisoma camini* (Slobodkina et al., 2012), the slightly halophilic *Microbulbifer variabilis* (Nishijima et al., 2009), *Microbulbifer* sp. ZGT114, which was isolated from a deep-sea brine pool in the Red Sea (KUJ81666), the alkaliphile *Geoalkalibacter ferrihydriticus* (Zavarzina et al., 2006), *Geobacter daltonii* and *Geobacter uraniireducens* (which were abundant and active in sediments undergoing uranium bioremediation; Shelobolina et al., 2008; Prakash et al., 2010), and *Geobacter metallireducens* (which can reduce plutonium and uranium; Lovley et al., 1991; Boukhalfa et al., 2007). Our analysis of the *H. seosinensis* transcriptomic response indicated that stress management was a crucial aspect of survival at high salinity. Lateral acquisitions of stress response genes that are present in stress-resistant bacteria further reinforce this statement and support the notion that LGT contributed to *H. seosinensis* adaptation to high salt environments.

## Conclusion

Although molecular information about halophilic fungi and algae has been accumulating in the past years, this study represents the first in-depth examination of gene expression in a halophilic bacterivorous protist. Overexpressed genes in *H. seosinensis* most likely allowed cells to adapt to a sustained higher level of ionic and oxidative stresses, and to acclimate the plasma membrane to enhanced hypersaline conditions. In addition, several gene duplication and LGT events potentially contributed to increase *H. seosinensis* salt adaptation over evolutionary time.

This study represents a first step in understanding *H. seosinensis* halophilicity at the molecular level. Since transcript level is not necessarily correlated with protein abundance or activity, and other control mechanisms in addition to transcriptional regulation occur in cells, further work, using approaches such as quantitative proteomics and determination of enzymatic activities, is required in order to validate the results and test the hypotheses presented herein. Moreover, future experiments designed to dissociate the influence on gene expression of physico-chemical and biological parameters co-varying with salinity (e.g., concentration of dissolved oxygen and food source quality) are greatly recommended.

## Author contributions

TH, AS, and AR designed the experiments. TH performed the work in laboratory and the data analyses. TH, AS, and AR wrote the manuscript.

### Conflict of interest statement

The authors declare that the research was conducted in the absence of any commercial or financial relationships that could be construed as a potential conflict of interest.
