# Supplementary material for: Adaptations to High Salt in a Halophilic Protist: Differential Expression and Gene Acquisitions through Duplications and Gene Transfers
Source: Front Microbiol. 2017 May 29;8:944. doi: 10.3389/fmicb.2017.00944 (PMC5447177; doi:10.3389/fmicb.2017.00944)
Supplement: Supplementary file 1 [file Table1.PDF]

Supplementary Table 1a. MMETSP taxa used to identify gene duplication candidates.

Species	MMETSP ID
Amphiprora sp.	MMETSP0724
Amphora coffeaeformis	MMETSP0316
Aplanochytrium sp.	MMETSP0954
Aristerstoma sp.	MMETSP0125
Aurantiochytrium limacinum	MMETSP0958
Aureococcus anophagefferens	MMETSP0914
Aureoumbra lagunensis	MMETSP0890
Bolidomonas pacifica	MMETSP0785
Cafeteria roenbergensis	MMETSP0942
Chaetoceros debilis	MMETSP0149
Corethron hystrix	MMETSP0010
Grammatophora oceanica	MMETSP0009
Heterosigma akashiwo	MMETSP0292
Ochromonas sp.	MMETSP0004
Odontella aurita	MMETSP0015
Odontella sinensis	MMETSP0160
Paraphysomonas imperforata	MMETSP0103
Pelagococcus subviridis	MMETSP0882
Pelagomonas calceolata	MMETSP0886
Percolomonas cosmopolitus	MMETSP0758
Pteridomonas danica	MMETSP0101
Skeletonema costatum	MMETSP0013
Thalassionema nitzschioides	MMETSP0156
Thraustochytrium sp.	MMETSP0198-0199

Supplementary Table 1b. Genomes used to identify gene duplication candidates.

Organisms	GenBank assembly accession
Aureococcus anophageferrens	GCA_000186865.1
Blastocystis hominis	GCA_000151665.1
Ectocarpus siliculosus	GCA_000310025.1
Nannochloropsis gadita	GCA_000240725.1
Phaeodactylum tricornutum	GCA_000150955.2
Phytophthora infestans	GCA_000142945.1
Phytophthora sojae	GCA_000149755.2
Phytophtora parasitica	GCA_000247585.2
Thalassiosira pseudonana	GCA_000149405.2
