# Supplementary material for: Adaptations to High Salt in a Halophilic Protist: Differential Expression and Gene Acquisitions through Duplications and Gene Transfers
Source: Front Microbiol. 2017 May 29;8:944. doi: 10.3389/fmicb.2017.00944 (PMC5447177; doi:10.3389/fmicb.2017.00944)
Supplement: Supplementary file 2 [file Table2.PDF]

Supplementary Table 2. Genomes used to survey gene duplication of P2X receptors.

Organisms	GenBank assembly accession
*Acanthamoeba castellanii**	GCF_000313135.1
*Acytostelium subglobosum**	GCF_000787575.1
*Aureococcus anophageferrens**	GCA_000186865.1
Blastocystis hominis	GCA_000151665.1
*Capsaspora owczarzaki**	GCF_000151315.2
*Dictyostelium fasciculatum**	GCF_000203815.1
*Dictyostelium purpureum**	GCF_000190715.1
Ectocarpus siliculosus	GCA_000310025.1
Nannochloropsis gadita	GCA_000240725.1
Phaeodactylum tricornutum	GCA_000150955.2
Phytophthora infestans	GCA_000142945.1
Phytophthora sojae	GCA_000149755.2
Phytophtora parasitica	GCA_000247585.2
*Salpingoeca rosetta**	GCF_000188695.1
Thalassiosira pseudonana	GCA_000149405.2

*: Genomes encoding P2X receptors.
