# Supplementary material for: Adaptations to High Salt in a Halophilic Protist: Differential Expression and Gene Acquisitions through Duplications and Gene Transfers
Source: Front Microbiol. 2017 May 29;8:944. doi: 10.3389/fmicb.2017.00944 (PMC5447177; doi:10.3389/fmicb.2017.00944)
Supplement: Supplementary file 3 [file Table3.PDF]

Supplementary Table 3. Differentially expressed genes coding for kinases in *Halocafeteria seosinensis.*

	Abundance (TPM)		EBSeq		DESeq2		VOOM-LIMMA	
ORF names	15% salt	30% salt	PPDE	Post fold change	Adjusted p-value	log_2FC	Adjusted p-value	log_2FC
**Sensory histidine kinases**								
m.13308	0.13	4.77	1.00	32.02	4.6E-06	3.86	0.0101	5.83
m.13214	8.44	44.21	1.00	4.48	0.0014	1.96	0.0266	2.38
**Mitogen-activated protein kinases**								
m.31471	0.87	22.34	1.00	18.79	2.0E-34	4.13	0.0006	4.23
m.81114	29.22	122.60	1.00	3.17	2.7E-09	1.64	0.0034	1.71
m.55540	3.09	12.17	1.00	3.23	0.0008	1.60	0.0227	1.68
m.51772	20.05	57.12	1.00	2.22	9.9E-06	1.14	0.0079	1.18
m.89580	2.72	8.29	1.00	2.38	4.4E-05	1.23	0.0130	1.21
m.4769	13.55	38.35	1.00	2.08	0.0048	1.03	0.0241	1.23
m.79532	3.34	9.64	1.00	2.13	0.0093	1.07	0.0273	1.31
**Calcium-dependent protein kinases**								
m.12706	0.10	3.53	1.00	19.63	7.2E-09	3.98	0.0030	4.44
m.37224	2.00	25.01	1.00	9.40	2.0E-43	3.21	0.0002	3.32
m.16106	1.15	10.89	1.00	7.08	4.6E-23	2.80	0.0006	2.92
m.24266	3.28	18.52	1.00	4.52	1.1E-14	2.14	0.0011	2.19
m.81494	5.17	14.26	1.00	2.19	0.0003	1.11	0.0168	1.13
**Calmodulin-dependent kinases**								
m.40160	6.14	23.26	1.00	2.99	4.4E-13	1.56	0.0019	1.58
**Other serine/threonine protein kinases**								
m.8926	0.06	19.38	1.00	185.19	3.7E-47	7.20	0.0004	7.65
m.80566	0.47	19.47	1.00	31.08	5.6E-78	4.92	0.0002	5.03
m.24240	48.72	232.76	1.00	3.67	1.1E-31	1.87	0.0004	1.92
m.88063	20.88	70.91	1.00	2.71	5.9E-06	1.41	0.0095	1.40
m.32208	36.37	95.80	1.00	2.07	1.1E-07	1.05	0.0045	1.09

Abbreviations: TPM, averaged transcripts per million (at 15% or 30% salt); PPDE, Probability of being Differentially Expressed, Post Fold Change, posterior fold change (30% over 15% salt); log_2FC, log_2 fold change (30% over 15% salt); NA, not available due to an extreme count outlier in one of the samples.
