# Supplementary material for: Adaptations to High Salt in a Halophilic Protist: Differential Expression and Gene Acquisitions through Duplications and Gene Transfers
Source: Front Microbiol. 2017 May 29;8:944. doi: 10.3389/fmicb.2017.00944 (PMC5447177; doi:10.3389/fmicb.2017.00944)
Supplement: Supplementary file 4 [file Table4.PDF]

Supplementary Table 4. Differentially expressed genes coding for transcription factors in *Halocafeteria seosinensis.*

	Abundance (TPM)		EBSeq		DESeq2		VOOM-LIMMA	
**ORF names**	**15% salt**	**30% salt**	**PPDE**	**Post fold change**	**Adjusted p-value**	**log_2FC**	**Adjusted p-value**	**log_2FC**
**BZIP transcription factors**								
m.26350	3.75	185.29	1.00	40.04	3.1E-67	5.19	0.0002	5.35
m.66567	11.10	117.89	1.00	8.14	1.3E-54	3.01	0.0002	3.09
m.26504	68.54	189.70	1.00	2.14	5.8E-05	1.08	0.013	1.09
**Sirtuins**								
m.20906	1.07	20.17	1.00	15.35	4.1E-17	3.74	0.0021	4.01
m.51236	27.68	138.54	1.00	3.76	4.9E-15	1.89	0.0011	1.99
**Heat shock transcription factor**								
m.36206	7.05	29.56	1.00	3.58	0.0029	1.70	0.021	1.94
m.87751	16.42	57.53	1.00	2.94	0.0007	1.49	0.017	1.60
m.51685	8.76	23.41	1.00	2.11	0.0007	1.06	0.023	1.05
**Myb superfamily of transcription factors**								
m.93181	21.02	121.64	1.00	4.71	NA	2.14	0.0057	2.29
m.93641	16.00	71.58	1.00	3.53	7.2E-22	1.81	0.0006	1.84
m.41207	5.87	21.85	1.00	2.82	1.3E-05	1.48	0.0066	1.62
m.83871	1.65	5.39	1.00	2.58	0.0054	1.32	0.033	1.41
m.63380	5.64	17.99	0.99	2.54	0.066	1.22	0.086	1.34
m.83876	17.41	8.57	1.00	0.38	1.3E-10	-1.36	0.0030	-1.34
m.53156	7.93	2.03	1.00	0.20	1.2E-10	-2.23	0.0019	-2.30
**AP2 domain-containing protein**								
m.1235	0.02	5.07	1.00	79.12	1.4E-17	5.92	0.0009	6.78
m.1227	9.01	167.52	1.00	14.10	4.4E-51	3.76	0.0002	3.90

Abbreviations: TPM, averaged transcripts per million; PPDE, Probability of being Differentially Expressed, Post Fold Change, posterior fold change (30% over 15% salt); log_2FC, log_2 fold change (30% over 15% salt); NA, not available due to an extreme count outlier in one of the samples.
