# Supplementary material for: Adaptations to High Salt in a Halophilic Protist: Differential Expression and Gene Acquisitions through Duplications and Gene Transfers
Source: Front Microbiol. 2017 May 29;8:944. doi: 10.3389/fmicb.2017.00944 (PMC5447177; doi:10.3389/fmicb.2017.00944)
Supplement: Supplementary file 5 [file Image1.PDF]

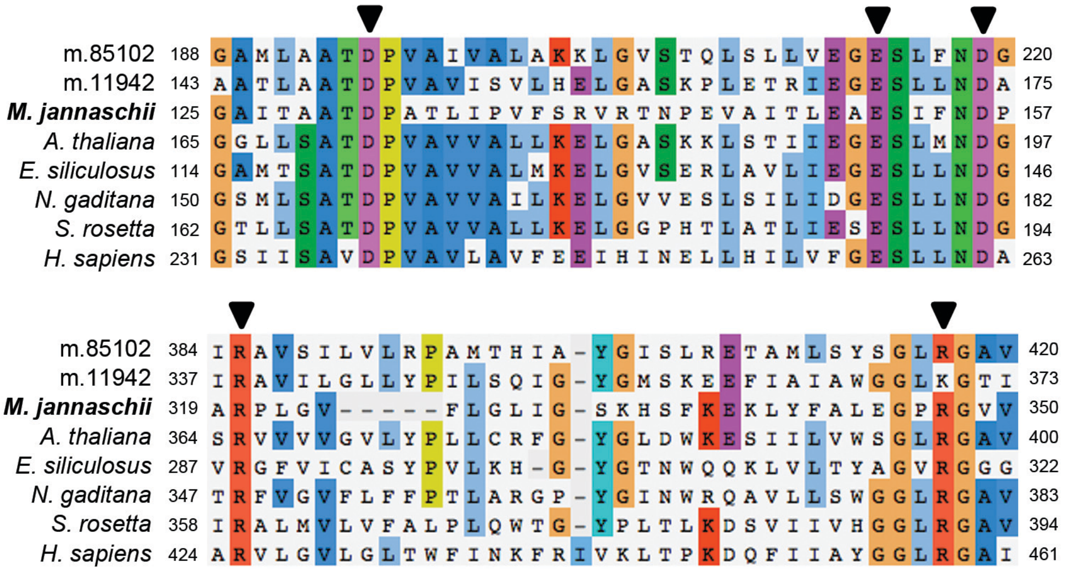

Supplementary Figure 1. Partial alignment of Na^+/H^+ antiporter protein sequences indicating conservation of crucial residues for catalytic activity (triangles) based on study of the *Methanoccocus jannaschii* enzyme (in bold, 4CZB; Hellmer et al., 2003). The alignment also includes sequences related to the *H. seosinensis* sequences (m.85102, m.11942) from *A. thaliana* (CCH26571.1), *Ectocarpus siliculosus* (CBJ26919.1), *Nannochloropsis gaditana* (EWM21970.1), *Salpingoeca rosetta* (XP_004995575.1) and from *Homo sapiens* (P19634.2).

Reference

Hellmer, J., Teubner, A., and Zeilinger, C. (2003). Conserved arginine and aspartate residues are critical for function of MjNhaP1, a Na+/H+ antiporter of *M. jannaschii*. *Febs Letters* 542(1-3)**,** 32-36. doi: 10.1016/s0014-5793(03)00332-6.
