# Supplementary material for: Adaptations to High Salt in a Halophilic Protist: Differential Expression and Gene Acquisitions through Duplications and Gene Transfers
Source: Front Microbiol. 2017 May 29;8:944. doi: 10.3389/fmicb.2017.00944 (PMC5447177; doi:10.3389/fmicb.2017.00944)
Supplement: Supplementary file 6 [file Image2.PDF]

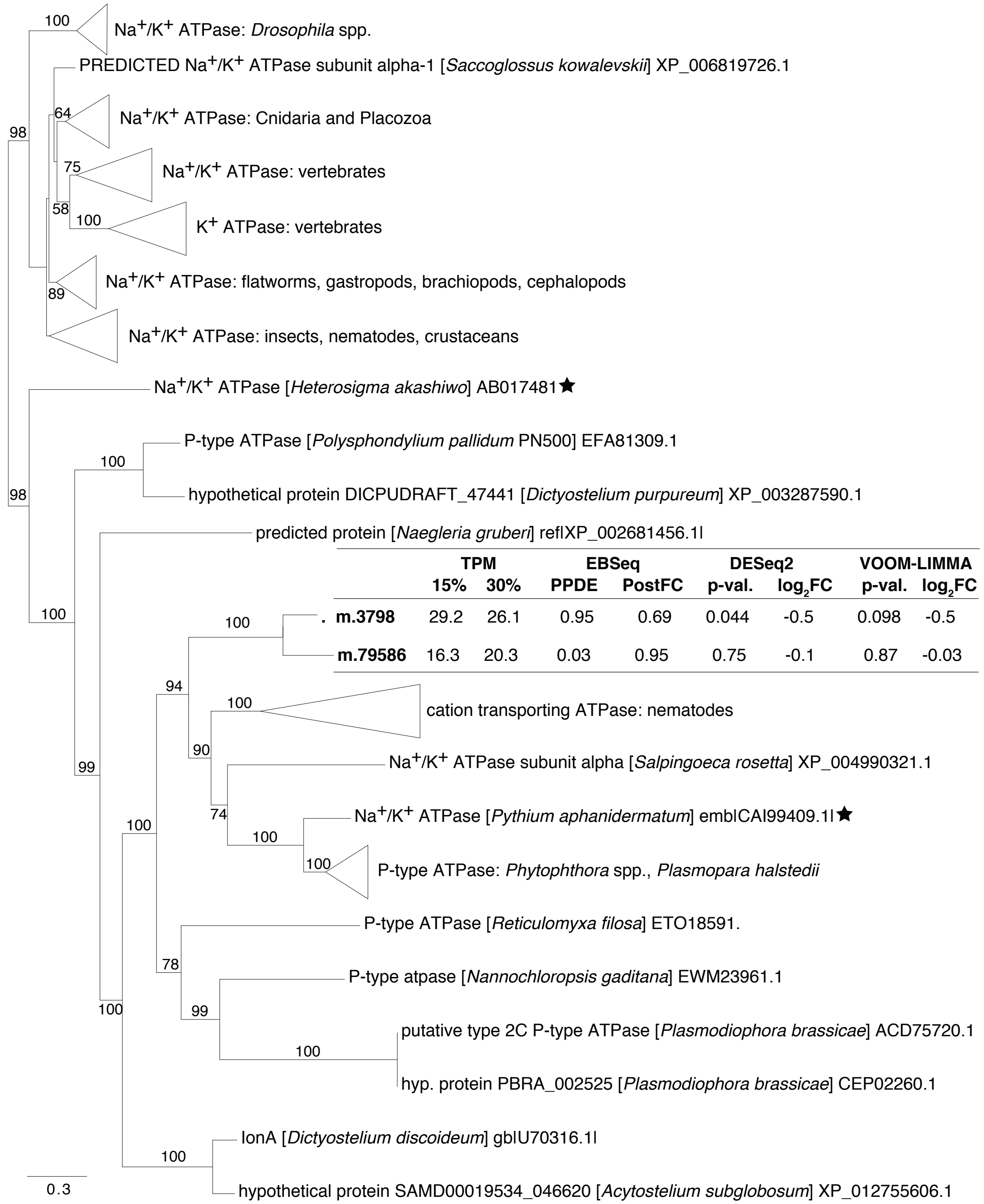

	TPM		EBSeq		DESeq2		VOOM-LIMMA	
	15%	30%	PPDE	PostFC	p-val.	log_2FC	p-val.	log_2FC
**m.3798**	29.2	26.1	0.95	0.69	0.044	-0.5	0.098	-0.5
**m.79586**	16.3	20.3	0.03	0.95	0.75	-0.1	0.87	-0.03

Supplementary Figure 2. Maximum-likelihood phylogenetic tree for gene duplication cluster encoding Na^+/K^+ ATPases. *Halocafeteria seosinensis* sequences (in bold) cluster inside a clade that contains sequences for ATPases shown to mediate Na^+ expulsion and K^+ uptake (stars). Bootstrap values >50% are indicated on branches. For *H. seosinensis* sequences, expression values are indicated as follows: TPM = averaged transcripts per million at 15% or 30% salt, PPDE = Posterior Probability of being Differentially Expressed and PostFC = Posterior Fold Change calculated by EBSeq, p-val. = adjusted p-value and log_2FC = log_2 fold change calculated either by DESeq2 or voom-limma. The scale bar indicates the expected substitutions/site.
