# Supplementary material for: Adaptations to High Salt in a Halophilic Protist: Differential Expression and Gene Acquisitions through Duplications and Gene Transfers
Source: Front Microbiol. 2017 May 29;8:944. doi: 10.3389/fmicb.2017.00944 (PMC5447177; doi:10.3389/fmicb.2017.00944)
Supplement: Supplementary file 7 [file Image3.PDF]

	TPM		EBSeq		DESeq2		VOOM-LIMMA	
	15%	30%	PPDE	PostFC	p-val.	log_2FC	p-val.	log_2FC
**m.21843**	47.3	25.6	1.00	0.4	7.4E-06	-1.2	0.010	-1.2
**m.39471**	25.7	47.1	0.040	1.5	0.21	0.5	0.20	0.6
**m.58046**	60.3	109.9	0.99	1.4	0.0042	0.5	0.038	0.6

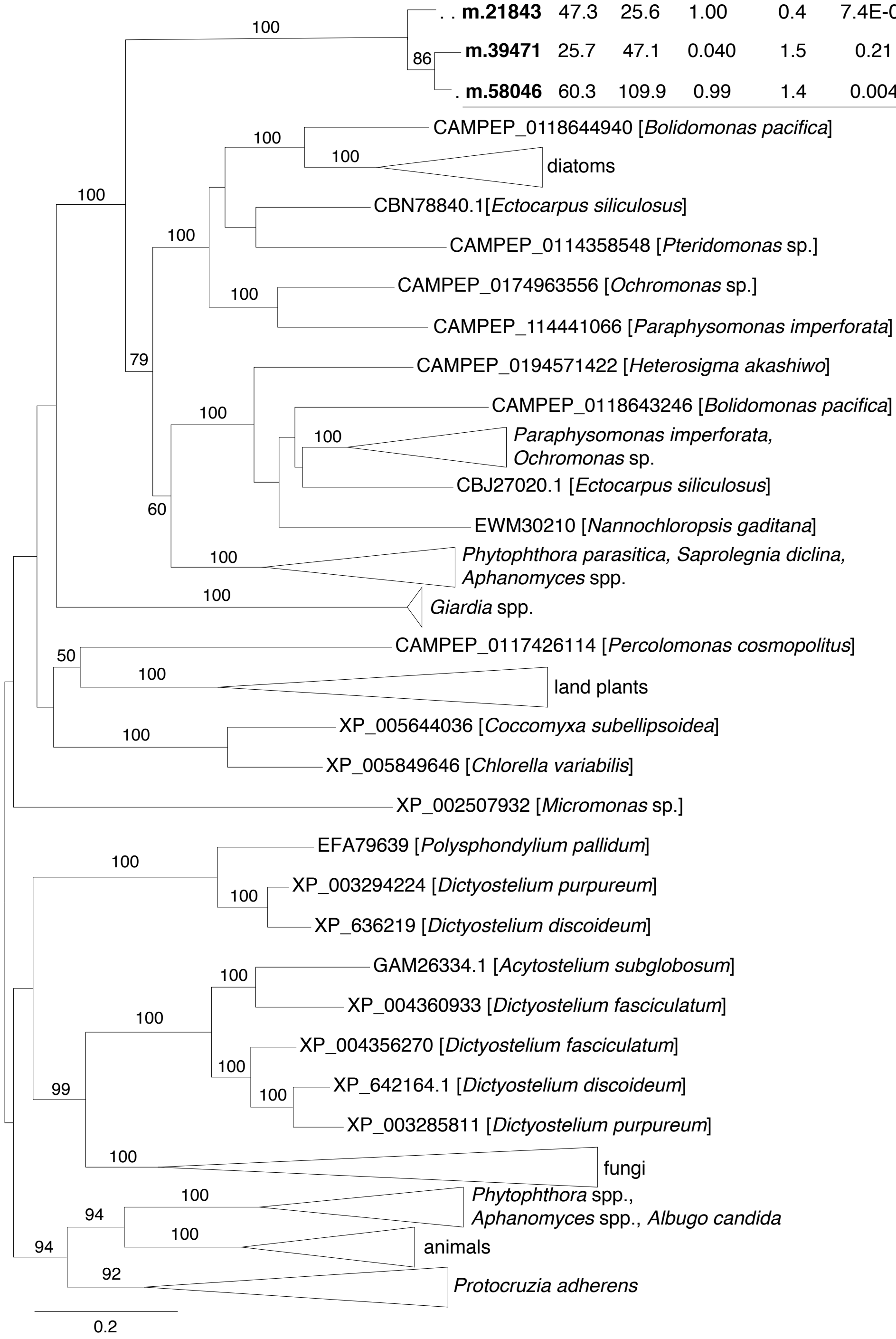

Supplementary Figure 3. Maximum-likelihood phylogenetic tree for gene duplication cluster encoding calcium-transporting ATPases. For *H. seosinensis* sequences (in bold), expression values are indicated: TPM = averaged transcript per million at 15% or 30% salt, PPDE = Posterior Probability of being Differentially Expressed and PostFC = Posterior Fold Change calculated by EBSeq, p-val. = adjusted p-value and log_2FC = log_2 fold change calculated either by DESeq2 or voom-limma. Bootstrap values (>50%) are indicated at branch nodes. The scale bar indicates the expected substitutions/site.
