# Supplementary material for: Adaptations to High Salt in a Halophilic Protist: Differential Expression and Gene Acquisitions through Duplications and Gene Transfers
Source: Front Microbiol. 2017 May 29;8:944. doi: 10.3389/fmicb.2017.00944 (PMC5447177; doi:10.3389/fmicb.2017.00944)
Supplement: Supplementary file 8 [file Image4.PDF]

	TPM		EBSeq		DESeq2		VOOM-LIMMA	
	15%	30%	PPDE	PostFC	p-val.	log_2FC	p-val.	log_2FC
**m.1669**	8.4	19.1	0.92	1.8	0.010	0.85	0.012	0.9
**m.57330**	13.0	17.3	0.047	1.0	0.98	0.01	0.75	0.1
**m.79946**	10.6	39.0	0.98	3.1	0.0028	1.53	0.0065	1.7

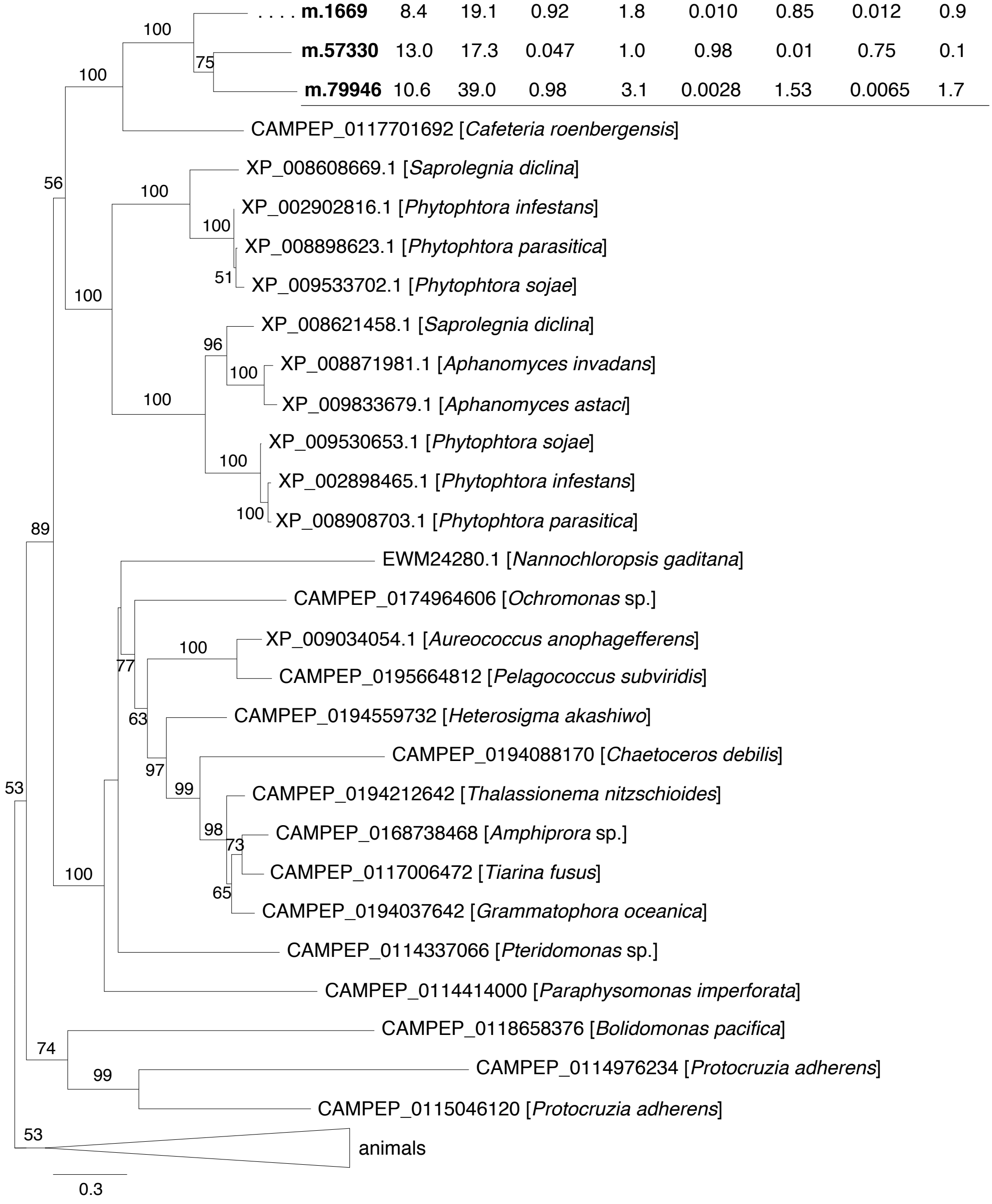

Supplementary Figure 4. Maximum-likelihood phylogenetic tree for gene duplication cluster encoding chloride channels. For *H. seosinensis* sequences (in bold), expression values are indicated: TPM = averaged transcript per million at 15% or 30% salt, PPDE = Posterior Probability of being Differentially Expressed and PostFC = Posterior Fold Change calculated by EBSeq, p-val. = adjusted p-value and log_2FC = log_2 fold change calculated either by DESeq2 or voom-limma. Bootstrap values (>50%) are indicated at branch nodes. The scale bar indicates the expected substitutions/site.
