# Supplementary material for: Adaptations to High Salt in a Halophilic Protist: Differential Expression and Gene Acquisitions through Duplications and Gene Transfers
Source: Front Microbiol. 2017 May 29;8:944. doi: 10.3389/fmicb.2017.00944 (PMC5447177; doi:10.3389/fmicb.2017.00944)
Supplement: Supplementary file 9 [file Image5.PDF]

	TPM		EBSeq		DESeq2		VOOM-LIMMA	
	15%	30%	PPDE	PostFC	p-val.	log_2FC	p-val.	log_2FC
**m.5273**	32.8	36.8	0.084	0.9	0.28	-0.2	0.38	-0.2
**m.6217**	83.4	147.2	1.00	1.4	4.7E-05	0.5	0.027	0.5

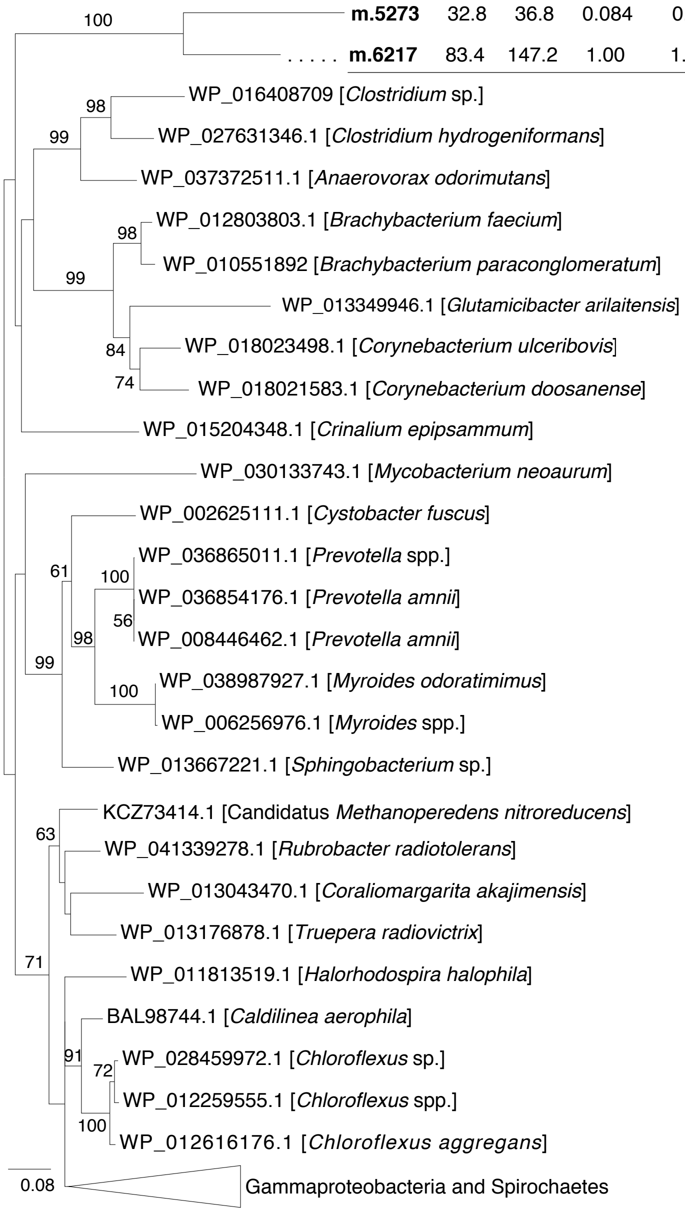

Supplementary Figure 5. Maximum-likelihood phylogenetic tree for gene duplication cluster encoding magnesium transporters. For *H. seosinensis* sequences (in bold), expression values are indicated: TPM = averaged transcript per million at 15% or 30% salt, PPDE = Posterior Probability of being Differentially Expressed and PostFC = Posterior Fold Change calculated by EBSeq, p-val. = adjusted p-value and log_2FC = log_2 fold change calculated either by DESeq2 or voom-limma. Bootstrap values (>50%) are indicated at branch nodes. The scale bar indicates the expected substitutions/site.
