# Supplementary material for: Adaptations to High Salt in a Halophilic Protist: Differential Expression and Gene Acquisitions through Duplications and Gene Transfers
Source: Front Microbiol. 2017 May 29;8:944. doi: 10.3389/fmicb.2017.00944 (PMC5447177; doi:10.3389/fmicb.2017.00944)
Supplement: Supplementary file 10 [file Image6.PDF]

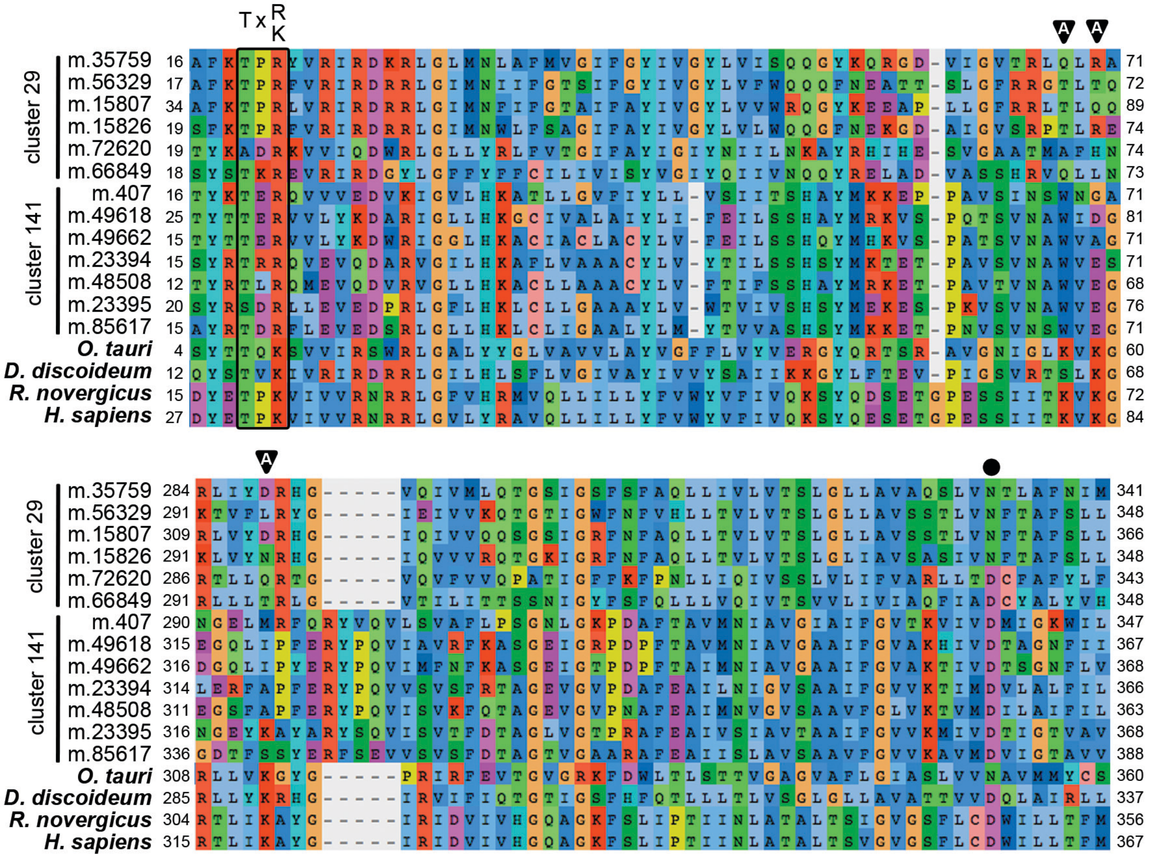

Supplementary Figure 6. Partial alignment of P2X receptor protein sequences indicating conservation of lysines involved in ATP binding ('A' in triangle) in reference sequences (in bold) from *O. tauri* (CEF98706.1), *D. discoideum* (XP_645378.1), *R. norvegicus* (P49653.1) and *H. sapiens* (Q9UBL9.1). These residues are not or partially conserved in the *H. seosinensis* sequences constituting gene duplication clusters 29 and 141. The protein kinase C recognition motif (Tx[K/R]) is boxed. The position indicated with a circle is essential for function and may be linked to calcium permeability.
