# Supplementary material for: Adaptations to High Salt in a Halophilic Protist: Differential Expression and Gene Acquisitions through Duplications and Gene Transfers
Source: Front Microbiol. 2017 May 29;8:944. doi: 10.3389/fmicb.2017.00944 (PMC5447177; doi:10.3389/fmicb.2017.00944)
Supplement: Supplementary file 11 [file Image7.PDF]

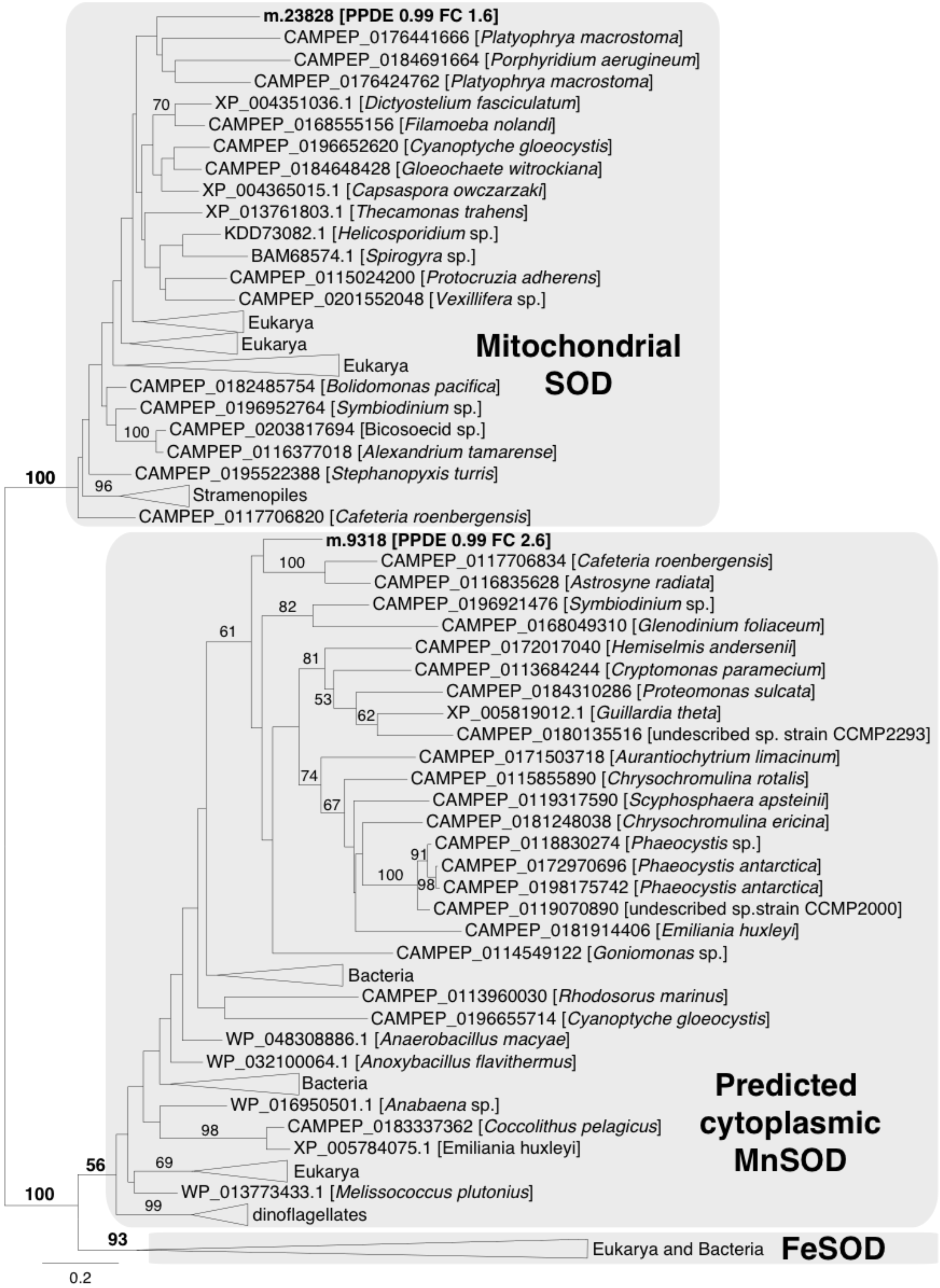

Supplementary Figure 7. Maximum-likelihood phylogenetic tree for superoxide dismutase (SOD) sequences showing three clades exclusively including; i) sequences annotated as mitochondrial SOD, ii) predicted cytoplasmic MnSOD sequences, for which no mitochondrial targeting peptide could be predicted (not applicable for bacterial sequences), and iii) sequences of described iron-dependent SOD (FeSOD). For *H. seosinensis* sequences (in bold), the Posterior Probability of being Differentially Expressed (PPDE) and the Fold Change (FC, 30% over 15% salt) is indicated. Bootstrap values (>50%) are indicated at branch nodes. The scale bar indicates the expected substitutions/site.
