# Supplementary material for: Adaptations to High Salt in a Halophilic Protist: Differential Expression and Gene Acquisitions through Duplications and Gene Transfers
Source: Front Microbiol. 2017 May 29;8:944. doi: 10.3389/fmicb.2017.00944 (PMC5447177; doi:10.3389/fmicb.2017.00944)
Supplement: Supplementary file 12 [file Image8.PDF]

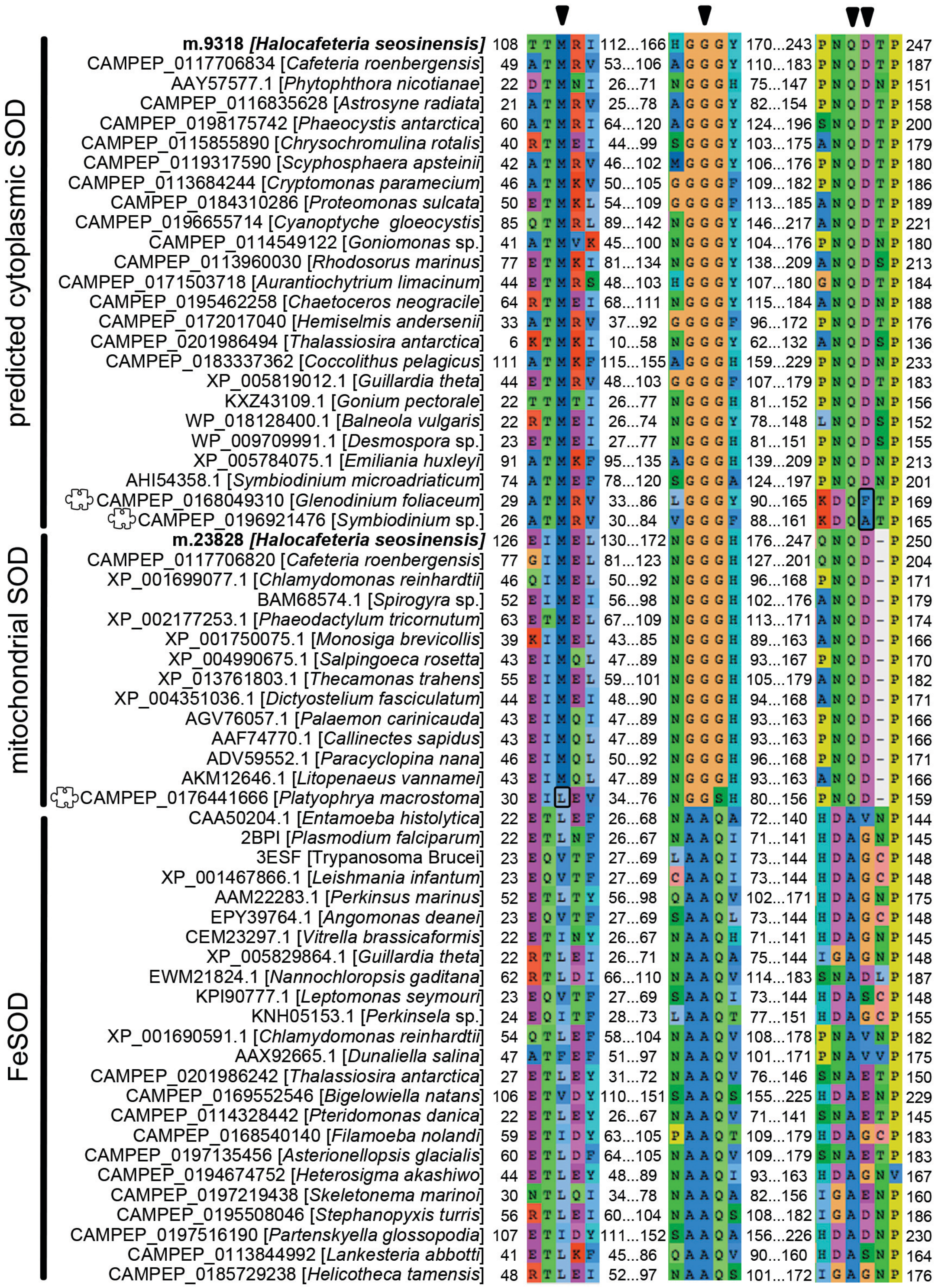

Supplementary Figure 8. Partial alignment of superoxide dismutase (SOD) sequences, showing residues exclusively conserved in cytoplasmic and mitochondrial manganese-dependent SOD (triangles) as described by Wintjens et al. (2004). Selected taxa are listed according to the clade in which they cluster in Figure 3.23. Puzzle pieces indicate the three sequences out of 205 that deviate from the expected motif for Mn-dependent SOD (boxed residues).

Reference

Wintjens, R., Noel, C., May, A.C.W., Gerbod, D., Dufernez, F., Capron, M., et al. (2004). Specificity and phenetic relationships of iron- and manganese-containing superoxide dismutases on the basis of structure and sequence comparisons. *Journal of Biological Chemistry* 279(10)**,** 9248-9254. doi: 10.1074/jbc.M312329200.
