# Supplementary material for: Adaptations to High Salt in a Halophilic Protist: Differential Expression and Gene Acquisitions through Duplications and Gene Transfers
Source: Front Microbiol. 2017 May 29;8:944. doi: 10.3389/fmicb.2017.00944 (PMC5447177; doi:10.3389/fmicb.2017.00944)
Supplement: Supplementary file 13 [file Image9.PDF]

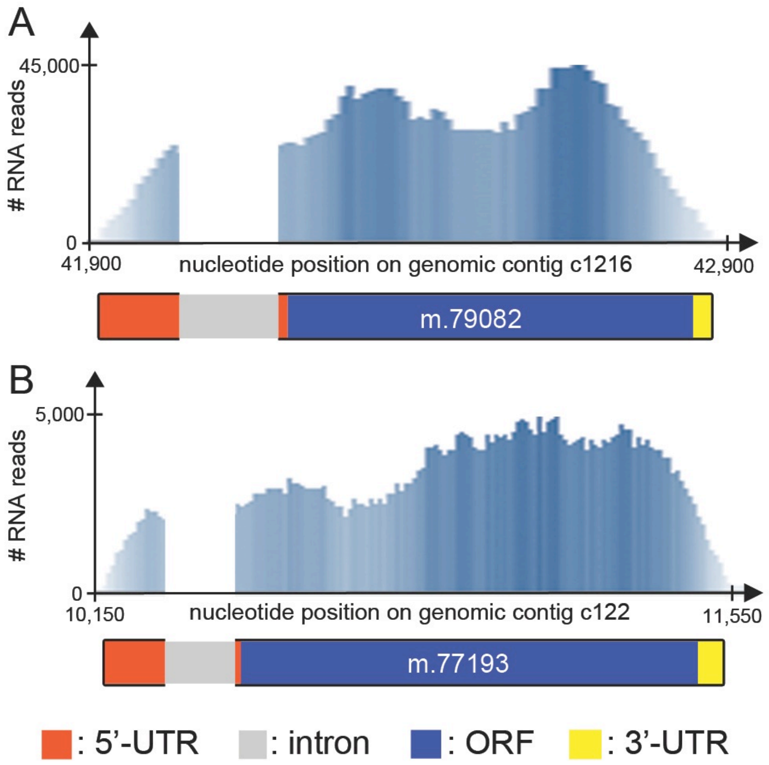

Supplementary Figure 9. Coverage of RNA-derived reads onto genomic contigs showing the presence of introns (in grey) in the 5'-untranslated regions (5'-UTR, in orange) of transcripts (shown under the plots) for open reading frames (ORF) m.79082 (related to peroxidase, panel A) and m.77193 (related to NADPH-dependent cinnamyl alcohol dehydrogenase, panel B). Forward reads generated from all replicates at 30% salt were mapped using TopHat2 2.0.13 onto genomic contigs c1216 (A; GenBank accession LVLI01000862) and c122 (B; LVLI01000090).
