# Supplementary material for: Adaptations to High Salt in a Halophilic Protist: Differential Expression and Gene Acquisitions through Duplications and Gene Transfers
Source: Front Microbiol. 2017 May 29;8:944. doi: 10.3389/fmicb.2017.00944 (PMC5447177; doi:10.3389/fmicb.2017.00944)
Supplement: Supplementary file 14 [file Image10.PDF]

A

	TPM 15%	TPM 30%	EBSeq PPDE	EBSeq PostFC	DESeq2 p-val.	DESeq2 log_2FC	VOOM-LIMMA p-val.	VOOM-LIMMA log_2FC
**m.21576**	306.6	1069.0	1.00	2.7	1.1E-04	1.4	0.017	1.4
**m.14941**	109.3	81.8	0.17	0.6	0.043	-0.8	0.14	-0.7

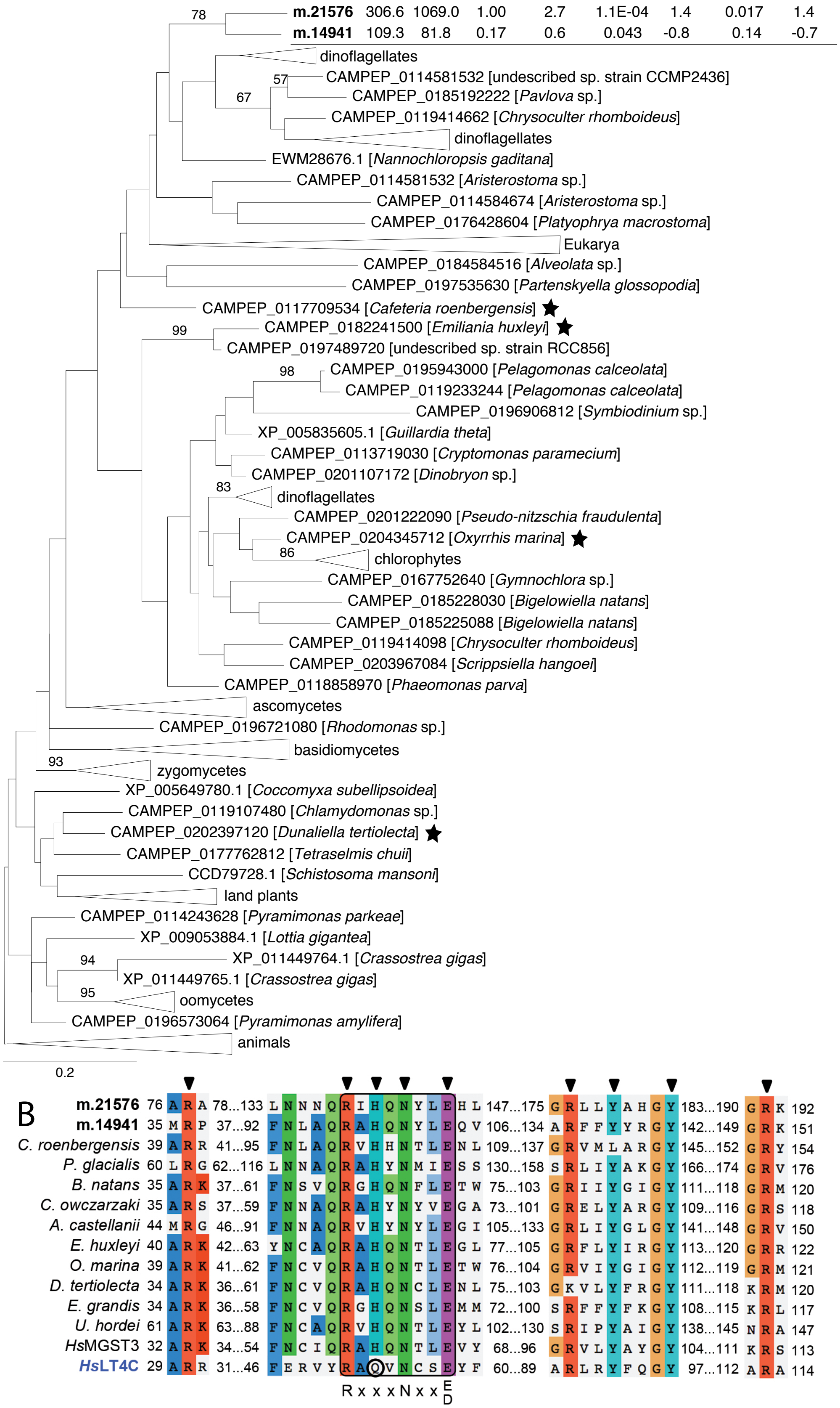

Supplementary Figure 10. Maximum-likelihood phylogenetic tree (A) and partial alignment (B) for gene duplication cluster encoding microsomal glutathione transferase. (A) For *H. seosinensis* sequences (in bold), expression values are indicated: TPM = averaged transcript per million at 15% or 30% salt, PPDE = Posterior Probability of being Differentially Expressed and PostFC = Posterior Fold Change calculated by EBSeq, p-val. = adjusted p-value and log_2FC = log_2 fold change calculated either by DESeq2 or voom-limma. Stars indicate sequences from species other than *H. seosinensis* that are included in the alignment in B. Bootstrap values (>50%) are indicated at branch nodes. The scale bar indicates the expected substitutions/site. (B) The alignment shows the MAPEG superfamily-wide conserved motif (Rx_3Nx_2[E/D], boxed) and residues that bind glutathione (triangles) in human leukotriene synthase (*Hs*LT4C in blue, 2PNO; Ago *et al.* 2007). Note that Q^{53} in *Hs*LT4C (circled) is conservatively substituted to His in microsomal glutathione S-transferase 3 (MGST3; Martinez Molina et al., 2008). Human MGST3 (*Hs*MGST3, AAB82609.1) is displayed as a reference in addition to *H. seosinensis* sequences (m.21576 and m.14941, in bold) and other related sequences from *Cafeteria roenbergensis* (CAMPEP_0117709534), *Polarella glacialis* (CAMPEP_0197963974), *Bigelowiella natans* (CAMPEP_0185225088), *Capsaspora owczarzaki* (XP_004364452.1), *Acanthamoeba castellanii* (XP_004333244.1), *Emiliania huxlei* (CAMPEP_0182241500), *Oxyrrhis marina* (CAMPEP_0204345712), *Dunalilella tertiolecta* (CAMPEP_0202397120), *Eucalyptus grandis* (XP_010062340.1) and *Ustilago hordei* (CCF50363.1).

References

Ago, H., Kanaoka, Y., Irikura, D., Lam, B.K., Shimamura, T., Austen, K.F., et al. (2007). Crystal structure of a human membrane protein involved in cysteinyl leukotriene biosynthesis. *Nature* 448(7153)**,** 609-612. doi: 10.1038/nature05936.

Martinez Molina, D., Eshaghi, S., and Nordlund, P. (2008). Catalysis within the lipid bilayer-structure and mechanism of the MAPEG family of integral membrane proteins. *Current opinion in structural biology* 18(4)**,** 442-449. doi: 10.1016/j.sbi.2008.04.005.
