# Supplementary material for: Adaptations to High Salt in a Halophilic Protist: Differential Expression and Gene Acquisitions through Duplications and Gene Transfers
Source: Front Microbiol. 2017 May 29;8:944. doi: 10.3389/fmicb.2017.00944 (PMC5447177; doi:10.3389/fmicb.2017.00944)
Supplement: Supplementary file 15 [file Image11.PDF]

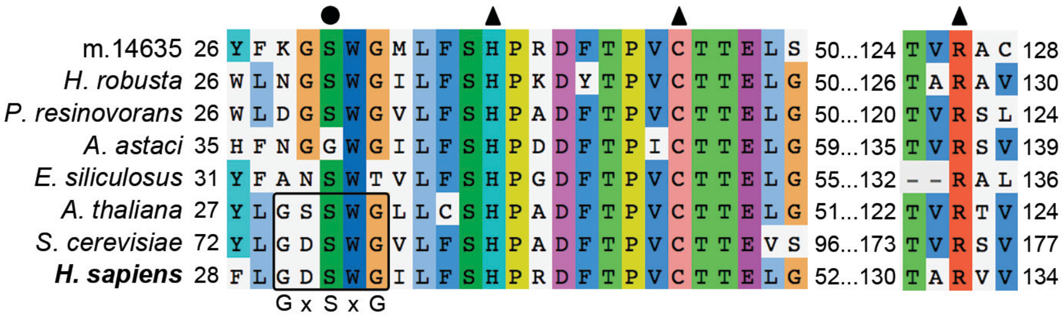

Supplementary Figure 11. Partial alignment of peroxiredoxin (Prdx) sequences showing the putative catalytic triad (triangles) based on study of human Prdx 6 crystal structure (in bold, NP_004896.1; Choi et al. 1998). Conservation of these residues in *H. seosinensis* sequence (m.14635) suggests that this enzyme is able to oxidize sn-2 fatty acyl group of phospholipids (peroxidase activity). Sequences from *Helobdella robusta* (XP_009018281.1) and *Pseudomonas resinovorans* (WP_028629099.1), like the *H. seosinensis* sequence, deviate from the lipase motif GxSxG (boxed), but include the putative serine catalytic residue (circle). Whether these enzymes can perform phospholipid hydrolysis in addition to the peroxidase activity, as commonly described in bifunctional Prdx 6, remains to be determined experimentally. For comparative purposes, sequences from *Aphanomyces astaci* (XP_009823721.1)*, Ectocarpus siliculosus* (CBN79130.1), *Arabidopsis thaliana* (NP_175247.1) and *S. cerevisiae* (AJQ03409.1) are included in the alignment.

Reference

Choi, H.J., Kang, S.W., Yang, C.H., Rhee, S.G., and Ryu, S.E. (1998). Crystal structure of a novel human peroxidase enzyme at 2.0 angstrom resolution. *Nature Structural Biology* 5(5)**,** 400-406. doi: 10.1038/nsb0598-400.
