# Supplementary material for: Adaptations to High Salt in a Halophilic Protist: Differential Expression and Gene Acquisitions through Duplications and Gene Transfers
Source: Front Microbiol. 2017 May 29;8:944. doi: 10.3389/fmicb.2017.00944 (PMC5447177; doi:10.3389/fmicb.2017.00944)
Supplement: Supplementary file 16 [file Image12.PDF]

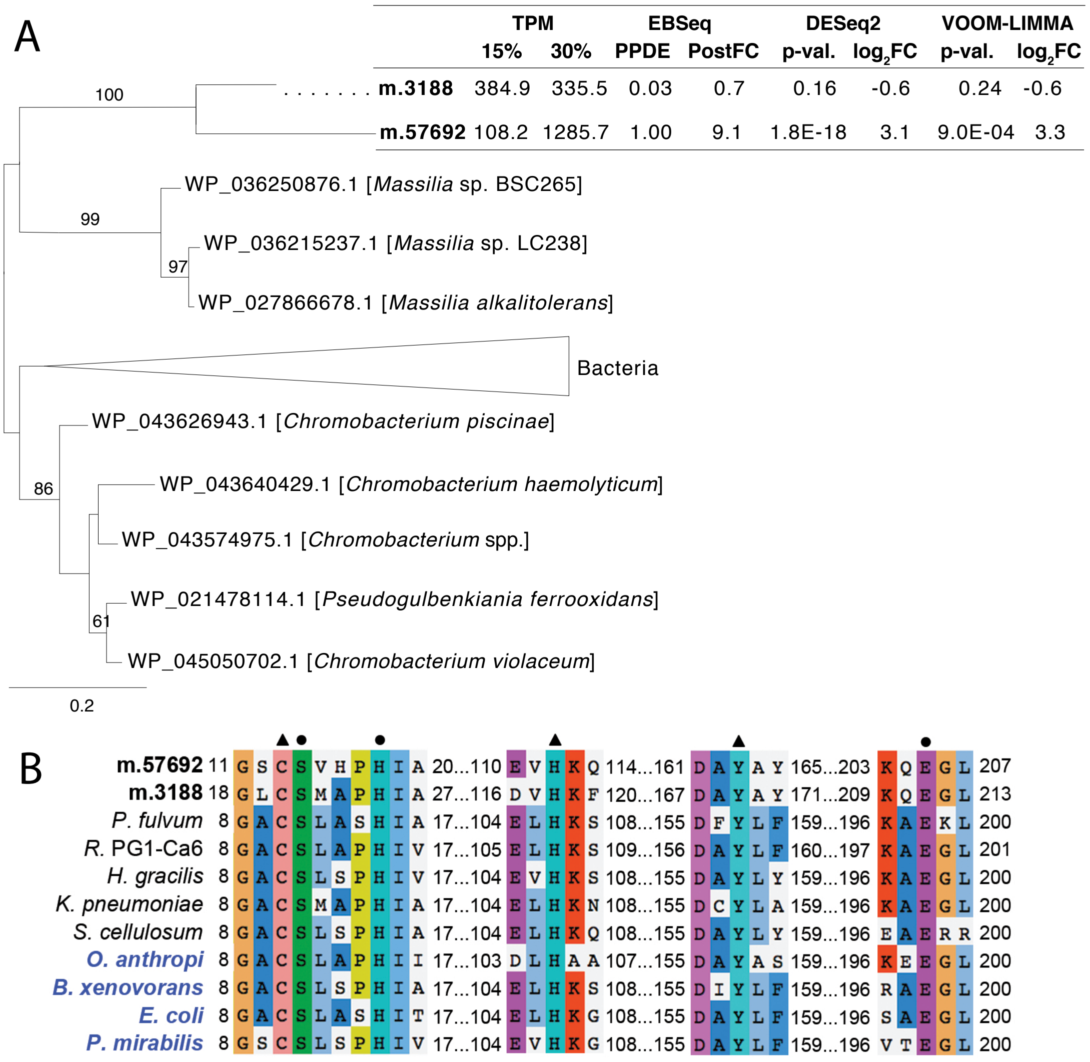

Supplementary Figure 12. Maximum-likelihood phylogenetic tree (A) and partial alignment (B) for gene duplication cluster encoding glutathione transferases of the Beta class. (A) For *H. seosinensis* sequences (in bold), expression values are indicated: TPM = averaged transcript per million at 15% or 30% salt, PPDE = Posterior Probability of being Differentially Expressed and PostFC = Posterior Fold Change calculated by EBSeq, p-val. = adjusted p-value and $\log_2$FC = $\log_2$ fold change calculated either by DESeq2 or voom-limma. Bootstrap values (>50%) are indicated at branch nodes. The scale bar indicates the expected substitutions/site. Note: This tree includes the 200 first BLAST hits from the NR database, which are of bacterial origin. However, eukaryotic homologs were detected in the MMETSP dataset with the closest sequence to *H. seosinensis* being 38% identical (compared to 50% identical for the first bacterial hit in the NR database). A phylogeny including all these eukaryote sequences was not resolvable, thus precluding a phylogenetic assessment of whether the *H. seosinensis* sequence was acquired by recent lateral gene transfer from bacteria. (B) The alignment shows conservation of residues interacting with the substrate (triangles) and residues essential for structural stability (circles) based on studies of enzymes (in blue) from *Ochrobactrum anthropi* (2NTO), *Burkholderia xenovorans* (2DSA), *Escherichia coli* (1N2A) and *Proteus mirabilis* (2PMT; Casalone *et al.* 1998; Allocati *et al.* 2000; Inoue et al. 2000; Federici *et al.* 2007; Federici *et al.* 2009). The alignment also includes *H. seosinensis* sequences (m.57692 and m.3188, in bold) and their closest related sequences in the NR database from *Phaeospirillum fulvum* (WP_021133055.1), Rhodocyclaceae bacterium PG1-Ca6 (AJP48267.1), *Hylemonella gracilis* (WP_035608673.1), *Klebsiella pneumoniae* (WP_040214185.1) and *Sorangium cellulosum* (WP_044968822.1).

References

Allocati, N., Casalone, E., Masulli, M., Polekhina, G., Rossjohn, J., Parker, M.W., et al. (2000). Evaluation of the role of two conserved active-site residues in Beta class glutathione S-transferases. *Biochemical Journal* 351**,** 341-346. doi: 10.1042/0264-6021:3510341.

Casalone, E., Allocati, N., Ceccarelli, I., Masulli, M., Rossjohn, J., Parker, M.W., et al. (1998). Site-directed mutagenesis of the *Proteus mirabilis* glutathione transferase B1-1 G-site. *Febs Letters* 423(2)**,** 122-124. doi: 10.1016/s0014-5793(98)00080-5.

Federici, L., Masulli, M., Bonivento, D., Di Matteo, A., Gianni, S., Favaloro, B., et al. (2007). Role of Ser^{11} in the stabilization of the structure of *Ochrobactrum anthropi* glutathione transferase. *Biochemical Journal* 403**,** 267-274. doi: 10.1042/bj20061707.

Federici, L., Masulli, M., Gianni, S., Di Ilio, C., and Allocati, N. (2009). A conserved hydrogen-bond network stabilizes the structure of Beta class glutathione S-transferases. *Biochemical and Biophysical Research Communications* 382(3)**,** 525-529. doi: 10.1016/j.bbrc.2009.03.052.

Inoue, H., Nishida, M., and Takahashi, K. (2000). Effects of Cys10 mutation to Ala in glutathione transferase from *Escherichia coli*. *Journal of Organometallic Chemistry* 611(1-2)**,** 593-595. doi: 10.1016/s0022-328x(00)00395-8.
