# Supplementary material for: Adaptations to High Salt in a Halophilic Protist: Differential Expression and Gene Acquisitions through Duplications and Gene Transfers
Source: Front Microbiol. 2017 May 29;8:944. doi: 10.3389/fmicb.2017.00944 (PMC5447177; doi:10.3389/fmicb.2017.00944)
Supplement: Supplementary file 17 [file Image13.PDF]

A

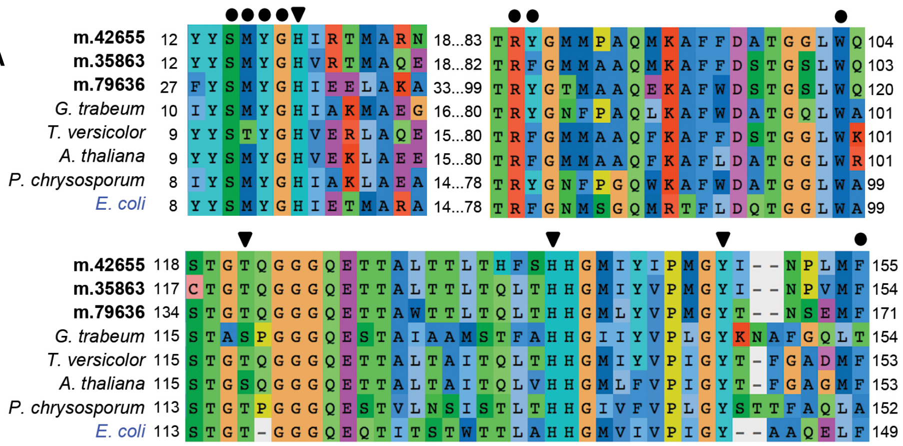

B

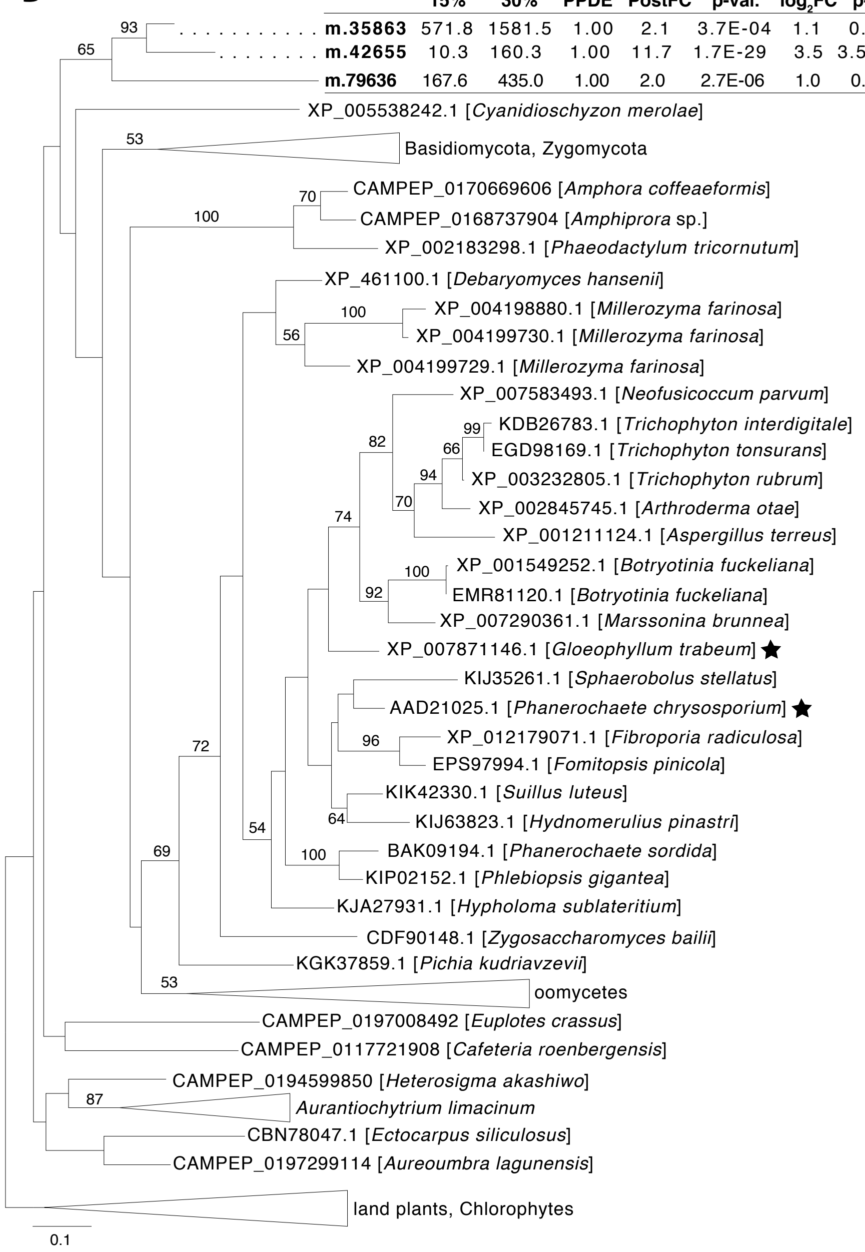

	TPM		EBSeq		DESeq2		VOOM-LIMMA	
	15%	30%	PPDE	PostFC	p-val.	log_2FC	p-val.	log_2FC
**m.35863**	571.8	1581.5	1.00	2.1	3.7E-04	1.1	0.021	1.1
**m.42655**	10.3	160.3	1.00	11.7	1.7E-29	3.5	3.5E-04	3.7
**m.79636**	167.6	435.0	1.00	2.0	2.7E-06	1.0	0.007	1.0

Supplementary Figure 13. Partial alignment (A) and maximum-likelihood phylogenetic tree (B) for gene duplication cluster encoding NAD(P)H : quinone oxidoreductases. (A) The alignment shows structural residues that shape the active-site cavity and contribute to orienting the substrate and cofactors (circles), and residues that interact through hydrogen bonds with the cofactor FMN (triangles) in *E. coli* WrbA (in blue, 3B6I), based on Andrade et al. (2007) and Wolfova et al. (2009). The alignment also includes *H. seosinensis* sequences (m.42655, m.35863 and m.79636, in bold), and sequences for which the enzymatic activity was characterized in *Gloeophyllum trabeum* (XP_007871146.1; Jensen *et al.* 2002), *Triphysaria versicolor* (AAG53945.1, Wrobel *et al.* 2002), *A. thaliana* (NP_200261.1, Laskowshi *et al.* 2002) and *Phanerochaete chrysosporium* (AAD21025.1, Brock *et al.* 1995). (B) For *H. seosinensis* sequences (in bold), expression values are indicated: TPM = averaged transcript per million at 15% or 30% salt, PPDE = Posterior Probability of being Differentially Expressed and PostFC = Posterior Fold Change calculated by EBSeq, p-val. = adjusted p-value and $\log_2$FC = $\log_2$ fold change calculated either by DESeq2 or voom-limma. Stars indicate sequences from characterized enzymes included in Supplementary Figure 17. Bootstrap values (>50%) are indicated at branch nodes. The scale bar indicates the expected substitutions/site.

References

Andrade, S.L.A., Patridge, E.V., Ferry, J.G., and Einsle, O. (2007). Crystal structure of the NADH : Quinone oxidoreductase WrbA from *Escherichia coli*. *Journal of Bacteriology* 189(24)**,** 9101-9107. doi: 10.1128/jb.01336-07.

Brock, B.J., Rieble, S., and Gold, M.H. (1995). Purification and characterization of a 1,4-benzoquinone reductase from the basidiomycete *Phanerochaete chrysosporium*. *Applied and Environmental Microbiology* 61(8)**,** 3076-3081.

Jensen, K.A., Ryan, Z.C., Wymelenberg, A.V., Cullen, D., and Hammel, K.E. (2002). An NADH : quinone oxidoreductase active during biodegradation by the brown-rot basidiomycete *Gloeophyllum trabeum*. *Applied and Environmental Microbiology* 68(6)**,** 2699-2703. doi: 10.1128/aem.68.6.2699-2703.2002.

Laskowski, M.J., Dreher, K.A., Gehring, M.A., Abel, S., Gensler, A.L., and Sussex, I.M. (2002). FQR1, a novel primary auxin-response gene, encodes a flavin mononucleotide-binding quinone reductase. *Plant Physiology* 128(2)**,** 578-590. doi: 10.1104/pp.010581.

Wolfova, J., Smatanova, I.K., Brynda, J., Mesters, J.R., Lapkouski, M., Kuty, M., et al. (2009). Structural organization of WrbA in apo- and holoprotein crystals. *Biochimica Et Biophysica Acta-Proteins and Proteomics* 1794(9)**,** 1288-1298. doi: 10.1016/j.bbapap.2009.08.001.

Wrobel, R.L., Matvienko, M., and Yoder, J.I. (2002). Heterologous expression and biochemical characterization of an NAD(P)H : quinone oxidoreductase from the hemiparasitic plant *Triphysaria versicolor*. *Plant Physiology and Biochemistry* 40(3)**,** 265-272.
