# Supplementary material for: Adaptations to High Salt in a Halophilic Protist: Differential Expression and Gene Acquisitions through Duplications and Gene Transfers
Source: Front Microbiol. 2017 May 29;8:944. doi: 10.3389/fmicb.2017.00944 (PMC5447177; doi:10.3389/fmicb.2017.00944)
Supplement: Supplementary file 18 [file Image14.PDF]

	TPM		EBSeq		DESeq2		VOOM-LIMMA	
	15%	30%	PPDE	PostFC	p-val.	log_2FC	p-val.	log_2FC
**m.27316**	7.2	12.2	0.10	1.4	0.32	0.4	0.34	0.4
**m.14436**	20.2	38.3	0.52	1.5	0.15	0.6	0.18	0.6
**m.82938**	4.2	38.3	1.00	7.1	1.4E-09	2.7	0.0086	2.8

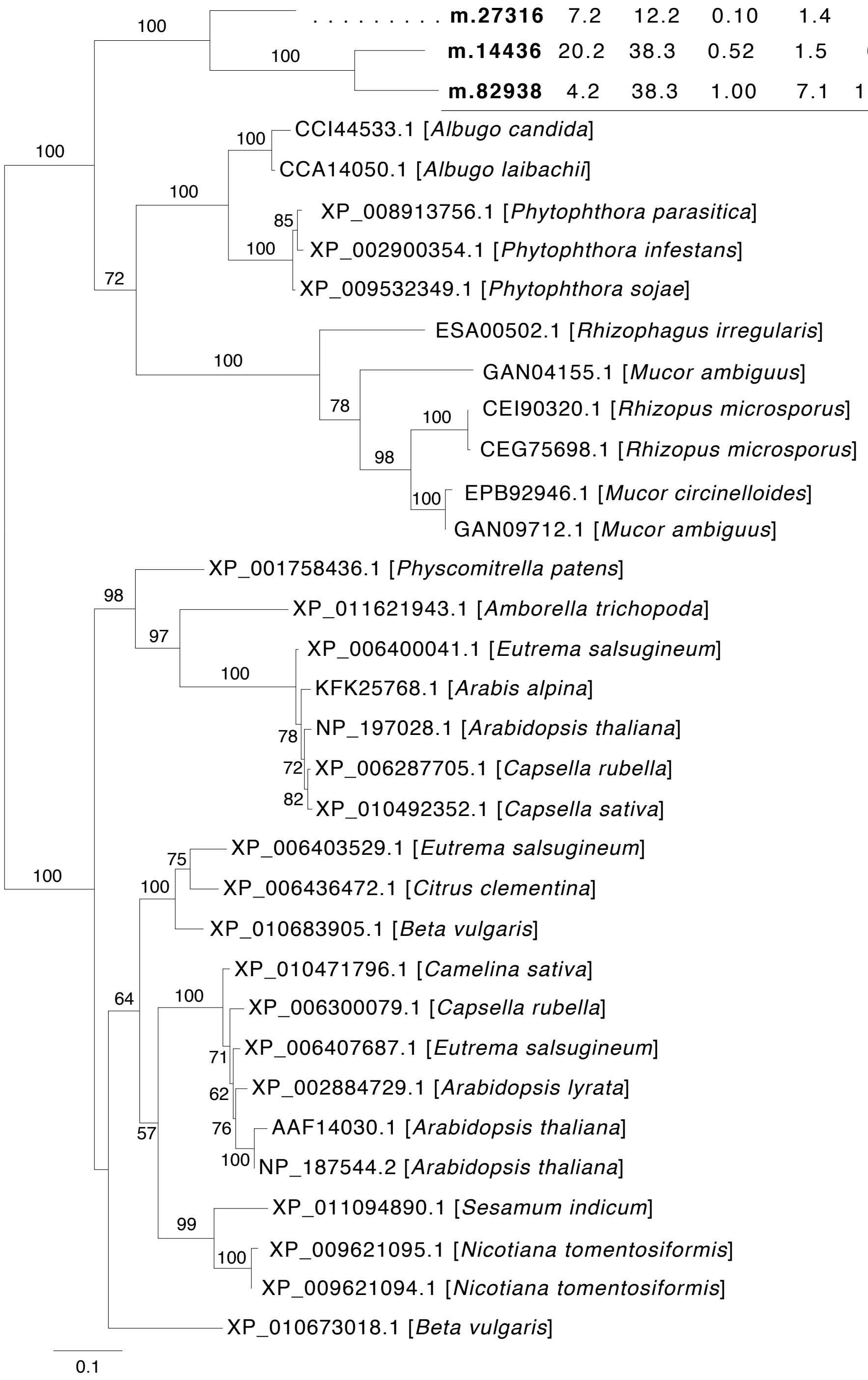

Supplementary Figure 14. Maximum-likelihood phylogenetic tree for gene duplication cluster encoding amino acid permeases. For *H. seosinensis* sequences (in bold), expression values are indicated: TPM = averaged transcript per million at 15% or 30% salt, PPDE = Posterior Probability of being Differentially Expressed and PostFC = Posterior Fold Change calculated by EBSeq, p-val. = adjusted p-value and log_2FC = log_2 fold change calculated either by DESeq2 or voom-limma. Bootstrap values (>50%) are indicated at branch nodes. The scale bar indicates the expected substitutions/site.
