# Supplementary material for: Adaptations to High Salt in a Halophilic Protist: Differential Expression and Gene Acquisitions through Duplications and Gene Transfers
Source: Front Microbiol. 2017 May 29;8:944. doi: 10.3389/fmicb.2017.00944 (PMC5447177; doi:10.3389/fmicb.2017.00944)
Supplement: Supplementary file 19 [file Image15.PDF]

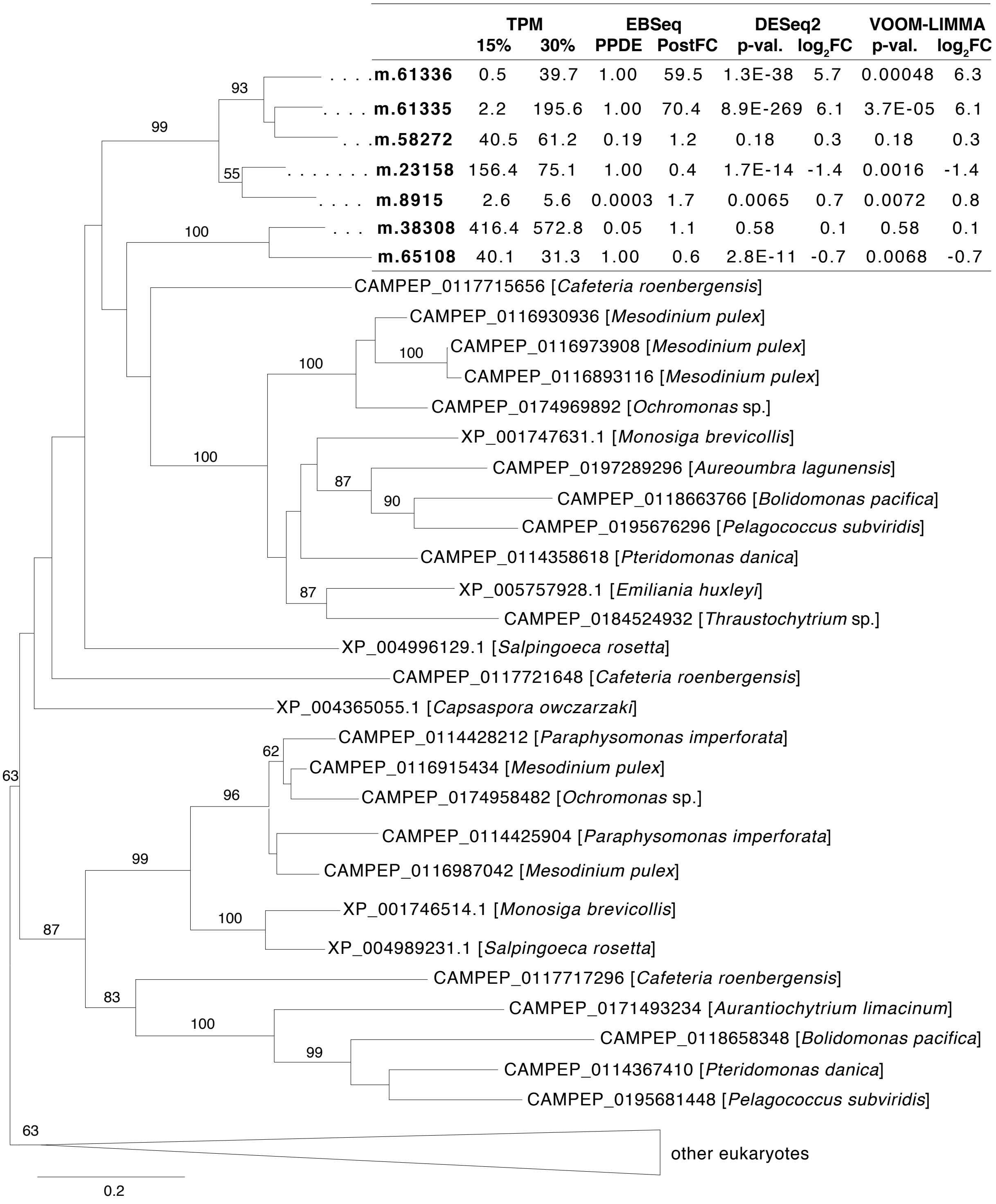

	TPM		EBSeq		DESeq2		VOOM-LIMMA	
	15%	30%	PPDE	PostFC	p-val.	log_2FC	p-val.	log_2FC
**m.61336**	0.5	39.7	1.00	59.5	1.3E-38	5.7	0.00048	6.3
**m.61335**	2.2	195.6	1.00	70.4	8.9E-269	6.1	3.7E-05	6.1
**m.58272**	40.5	61.2	0.19	1.2	0.18	0.3	0.18	0.3
**m.23158**	156.4	75.1	1.00	0.4	1.7E-14	-1.4	0.0016	-1.4
**m.8915**	2.6	5.6	0.0003	1.7	0.0065	0.7	0.0072	0.8
**m.38308**	416.4	572.8	0.05	1.1	0.58	0.1	0.58	0.1
**m.65108**	40.1	31.3	1.00	0.6	2.8E-11	-0.7	0.0068	-0.7

Supplementary Figure 15. Maximum-likelihood phylogenetic tree for gene duplication cluster encoding carboxy-peptidases. For *H. seosinensis* sequences (in bold), expression values are indicated: TPM = averaged transcript per million at 15% or 30% salt, PPDE = Posterior Probability of being Differentially Expressed and PostFC = Posterior Fold Change calculated by EBSeq, p-val. = adjusted p-value and log_2FC = log_2 fold change calculated either by DESeq2 or voom-limma. Bootstrap values (>50%) are indicated at branch nodes. The scale bar indicates the expected substitutions/site.
